# Epstein–Barr virus EBNA2 phase separation regulates cancer‐associated alternative RNA splicing patterns

**DOI:** 10.1002/ctm2.504

**Published:** 2021-08-09

**Authors:** Qiu Peng, Lujuan Wang, Jia Wang, Can Liu, Xiang Zheng, Xiaoyue Zhang, Lingyu Wei, Zhengshuo Li, Yangge Wu, Yuqing Wen, Pengfei Cao, Qianjin Liao, Qun Yan, Jian Ma

**Affiliations:** ^1^ Hunan Key Laboratory of Cancer Metabolism Hunan Cancer Hospital and the Affiliated Cancer Hospital of Xiangya School of Medicine Central South University Changsha China; ^2^ Cancer Research Institute and School of Basic Medical Science Central South University Changsha China; ^3^ Key Laboratory of Carcinogenesis and Cancer Invasion of the Chinese Ministry of Education, NHC Key Laboratory of Carcinogenesis, Hunan Key Laboratory of Nonresolving Inflammation and Cancer Hunan Key Laboratory of Translational Radiation Oncology Hunan Cancer Hospital and The Affiliated Cancer Hospital of Xiangya School of Medicine, Central South University Changsha China; ^4^ Department of Immunology Department of Pathology Heping Hospital, Changzhi Medical College Changzhi China; ^5^ Department of Pathology Affiliated Hospital of Guilin Medical University Guilin China; ^6^ Department of Clinical Laboratory Department of Hematology Xiangya Hospital Central South University Changsha China

Dear Editor,

Alternative splicing of pre‐mRNAs is a significant mRNA maturation process that increases RNA and protein diversity in eukaryotes.^1^, ^2^, ^3^ The Epstein–Barr Virus (EBV)‐encoded nuclear antigen 2 (EBNA2) is a multifunctional transcriptional activator and is essential for EBV‐induced cell transformation through activating multiple genes expression.^4^, ^5^, ^6^ We recently revealed that EBNA2 activates cellular genes transcription by phase separation.^7^

To investigate the components of these phase separation nuclear puncta that interact with EBNA2, we used mass spectrometry to analyze the EBNA2 co‐immunoprecipitates and found that 193 cellular proteins were most likely to interact with EBNA2 (Figure 1A and Table S1). Bioinformatics analysis showed that EBNA2‐interacted proteins are mainly enriched in the RNA splicing pathway (Figure 1B, Figures S1A and S1B). Among the 193 EBNA2‐interacted proteins, splicing factors SRSF1 and SRSF7's peptide patterns have a considerable high score through mass spectrometry (Figure 1C,D). Reciprocal co‐immunoprecipitation and immunofluorescence analysis confirmed that EBNA2 interacts with SRSF1 and SRSF7 (Figure 1E–H).

**FIGURE 1:**
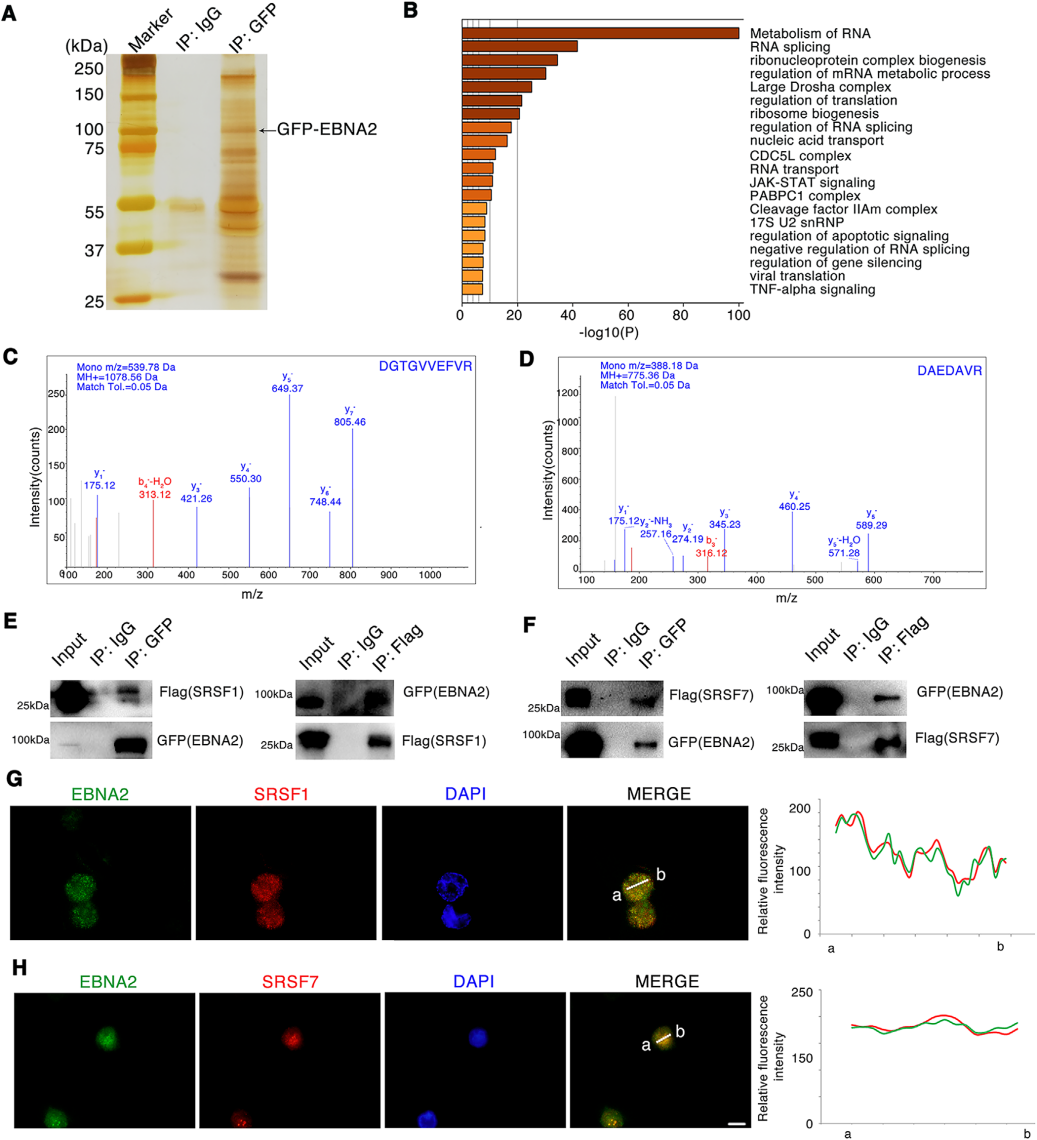
EBNA2 interacts with components of the splicing machinery. (A) Whole‐cell extracts from HEK293 cells expressing GFP‐EBNA2 were subjected to affinity purification with anti‐GFP antibody, and the enriched proteins were collected and subjected to SDS‐PAGE electrophoresis and silver staining. The bands were retrieved and analyzed by mass spectrometry. (B) Enriched KEGG pathways related to the EBNA2‐interacted proteins. (C, D) SRSF1(C) and SRSF7 (D) were included in the EBNA2‐complex through co‐IP combined with mass spectrometry. Representative images from mass spectrometry. (E, F) Interaction between EBNA2 with SRSF1 (E) or SRSF7 (F) after transient expression of the indicated expression plasmids in HEK293 cells for 48 h. Western blotting of cell lysates subjected to co‐IP with anti‐Flag or anti‐GFP. (G, H) Co‐localization of endogenous EBNA2 and SRSF1 (G) and SRSF7 (H) in Raji cells as assessed with immunofluorescence staining. Raji cell is an EBV‐positive cell line. The EBNA2 and SRSF1, SRSF7 signals were measured by ImageJ software (right panel). Scale bar, 20 μm

To identify whether EBNA2 is linked to pre‐mRNA alternative splicing processes, we analyzed the differentially expressed genes and transcripts induced by EBNA2 by integrating third‐generation SMRT sequencing and second‐generation RNA‐sequencing (Figure 2A). EBNA2 induced 6071 differentially expressed full‐length transcripts and 929 genes (Figures S2A, S3A, and S4A), as well as 2752 differentially alternative splicing (DAS) events (Figure 2B, C). When comparing the EBNA2‐induced DAS genes with the differentially expressed genes (DEG), an overlap of only 40 genes (1.1%) was obtained (Figure S2B). Functional GO categories of metabolic processes are observed in both the DAS genes and the DEG (Figure 2D, E). EBNA2‐induced DAS genes were enriched for functional categories of defense responses and tumor‐related signaling pathways (Figure S3B, C), whereas EBNA2‐induced DEGs were enriched for functional categories related to mRNA processing and DNA repair (Figure S4B–D).

**FIGURE 2:**
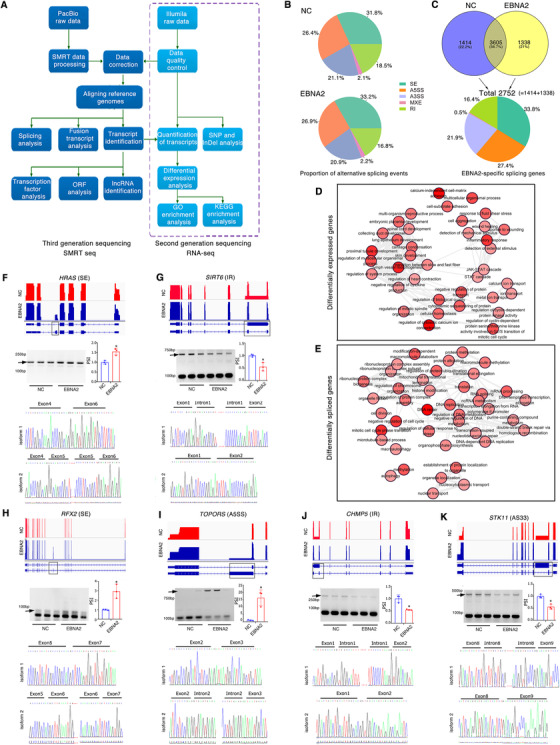
Identification of EBNA2‐regulated alternative splicing events. (A) Workflow for sequencing and analysis of EBNA2‐treated specimens. Left is for the third generation sequencing (SMRT); right is for the second generation sequencing (RNA‐seq). (B) The proportion of alternative splicing events in EBNA2‐expression or negative control (NC) cells: SE, skipped exons; A3SS, alternative 3′ splice sites; A5SS, alternative 5′ splice sites; MXE, mutually exclusive exons, RI, intron retention. (C) Quantification of EBNA2‐regulated alternative splicing events in each category (NC and EBNA2) (*up panel*). 2752 events are specifically related to EBNA2 regulation (*bottom panel*). (D, E) REVIGO plots (http://revigo.irb.hr) of GO enrichment clusters of differentially expressed genes (D), or differentially spliced genes (E) induced by EBNA2 expression. Each circle represents a significant GO category but only groups of highest significance are labeled. (F–K) *Up panel*, normalized sequencing coverage data are shown in each panel. Maps of EBNA2‐specific isoforms shown with exon arrangement and alternative splicing sites highlighted (black rectangle). The scale is the same on each track. Relative expression for each gene was calculated based on the normalized RNA‐seq read count. *Middle panel*, a black arrow marks the position of the EBNA2‐regulated alternative splicing isoforms. RT‐PCR was performed on 3 biological replicates, and relative expressions for the isoforms are indicated in the right. *Bottom panel*, sanger sequencings show novel junctions of EBNA2‐specific isoforms

The EBNA2‐induced DAS genes are involved in tumorigenesis. We identified 138 tumor suppressor genes and 86 oncogenes among the DAS genes by means of the TSGene database and ONGene database (Figure S5A, B). Through gene set enrichment analysis of the DAS genes, we found gene sets associated with cancer‐related pathways are strongly enriched in EBNA2‐overexpressing cells compared with NC cells (Figure S6).

To further verify the accuracy of the DAS profiles, we analyzed 12 EBNA2‐induced alternative splicing isoforms by Integrative Genomics Viewer tools and observed that all results from RT‐PCR and Sanger sequencing coincided with alternative splicing changes identified by SMRT‐seq and RNA‐seq (Figures 2F–K and Figure S7).

Among EBNA2‐regulated alternative splicing isoforms, we observed that the Percentage Spliced In (PSI) value, (which represents the mRNA percentage of one indicated isoform) of *MPPE1* exon 11 inclusion splice variants (*MPPE1* 11+, transcript variant 3) was significantly reduced by EBNA2 (Figure 3A). *MPPE1* 11‐ isoform (transcript variant 1) was further confirmed by RT‐PCR, PAGE analysis, and Sanger sequencing (Figure 3B, C). Interestingly, we noticed that the inclusion of exon 11 leads to the introduction of a premature termination codon (Figure 3D), and the *MPPE1* transcript variant 3 is a non‐coding RNA (Figure 3E, confirmed on the UCSC website). Next, we constructed an *MPPE1* exon11 minigene reporter system (Figure 3F). As shown in Figure 3G, EBNA2 expression causes a significant increase in EGFP or RFP signal compared to the NC, suggesting that EBNA2 promotes exon 11 exclusion.

**FIGURE 3:**
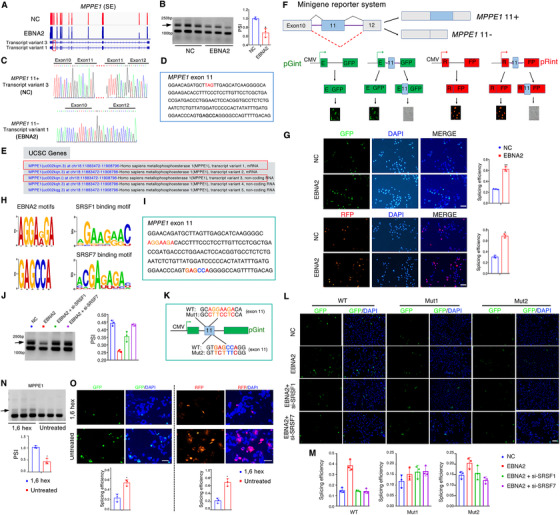
EBNA2‐induced alternative splicing of *MPPE1* is regulated by splicing factors SRSF1/SRSF7 and phase separation. (A) Normalized sequencing coverage data of two *MPPE1* transcript variants. Maps of EBNA2‐specific isoforms shown with exon arrangement and alternative splicing sites highlighted (red rectangle). The scale is the same on each track. Relative expression for *MPPE1* was calculated based on the normalized RNA‐seq read count. (B) RT‐PCR analysis of *MPPE1* transcripts demonstrating a change in splicing pattern in HEK293 cells after EBNA2 expression. A black arrow marks the position of the EBNA2‐regulated alternative isoform, and relative expressions for the isoforms are indicated on the right. (C) Sanger sequencings show novel junctions of *MPPE1* in EBNA2 expressed cells (bottom panel). (D) The nucleic acid sequence of the human *MPPE1* exon 11. The red font indicates the introduced premature stop codon. (E) Encoding information of multiple *MPPE1* transcript variants displayed in the UCSC database (http://genome.ucsc.edu). (F) The diagram presents of *MPPE1* exon 11 splicing minigene reporter system. (G) Minigene reporter system for detecting *MPPE1* exon11. The fluorescence signal in the EGFP or RFP channel represents exon 11 splicing efficiency in NC and EBNA2 expressed cells. Quantification of splicing efficiency by measuring the relative expression of intact EGFP or RFP transcript. Scale bar, 100 μm. (H) Left: “EBNA2 motifs” predicted by the MEME Suit (http://meme‐suite.org) through analyzing the sequences of validated EBNA2‐regulated alternative splicing events. Right: SRSF1 and SRSF7 binding motifs identified in the DeepBind database (http://tools.genes.toronto.edu/deepbind/). (I) The nucleic acid sequence of the human *MPPE1* exon 11. The color font indicates the SRSF1 and SRSF7 binding motifs. (J) A black arrow marks the position of the *MPPE1* exon 11 alternative isoforms. RT‐PCR was performed on 3 biological replicates, and relative expressions for the isoforms are indicated in the right. (K) The diagram presents the *MPPE1* exon 11 composition and the corresponding sequence of the wild‐type (WT) splicing reporter and derived mutants of the binding motifs of SRSF1 and SRSF7 on *MPPE1* exon 11. (L) Minigene reporter system for detecting *MPPE1* exon 11 and derived mutants; the fluorescence signal in the EGFP channel represents exon 11 splicing efficiency in the above‐treated cells. Scale bar, 100 μm. (M) Quantification of splicing efficiency by measuring the relative expression of intact EGFP transcript. (N) RT‐PCR analysis of *MPPE1* transcripts demonstrating a change in splicing pattern in over‐expressed EBNA2 HEK293 cells after treatment with 0.5% 1,6 hexanediol for 2 h. A black arrow marks the position of the EBNA2‐regulated alternative isoform, and relative expressions for the isoforms are indicated in the bottom panel. (O) Minigene reporter system for detecting *MPPE1* exon 11. The fluorescence signal in the EGFP or RFP channel represents exon 11 splicing efficiency before and after treatment with 0.5% 1,6 hexanediol for 2 h in EBNA2 expressed cells. Quantification of splicing efficiency by measuring the relative expression of intact EGFP or RFP transcript is indicated in the bottom panel. Data are presented as mean ± SD

We discovered over‐expression of EBNA2 had no effect on the cellular spliceosome components mRNA levels by analyzing RNA‐seq data (Figure S8). To determine the mechanisms by which EBNA2 regulates alternative splicing of the pre‐*MPPE1* gene, we used the MEME Suit to analyze the sequences and found two consensus motif sequences with predicted “EBNA2 motifs,” which almost overlapped with the SRSF1 and SRSF7 binding motifs (Figure 3H, I) confirmed in the DeepBind database. RT‐PCR and minigene reported system revealed that knockdown of SRSF1 or SRSF7 rescued the EBNA2‐induced *MPPE1* exon 11 exclusion (Figure 3J–M). To investigate whether the phase separation capacity of EBNA2 is associated with splicing, we treated EBNA2‐overexpressed cells with 1,6‐hexanediol (an aliphatic alcohol that can disrupt phase separation) and found it caused a significant increase in the expressions of *MPPE1* exon 11 inclusion splice variants (Figure 3N, O). These results suggest that EBNA2 controls *MPPE1* splicing through phase separation.

Given that EBV was involved in multiple tumors progression, we speculated that EBNA2‐induced *MPPE1* transcript variant 1 might also function in cancer development. Through analyzing the Oncomine database, we noticed significantly elevated *MPPE1* expression in more than ten kinds of tumor tissues (Figure 4A and Figure S9). *MPPE1* expression was significantly higher in gastric cancer samples compared to non‐cancer controls (Figure 4B, confirmed in The Cancer Genome Atlas (TCGA) database). In addition, gastric cancer patients (GSE62254) with higher levels of *MPPE1* expression showed shorter overall survival (Figure 4C). We next transfected *MPPE1* 11‐ expression vector into AGS or MKN‐7 cells that have lower endogenous *MPPE1* 11‐ expression than other gastric cancer cell lines (Figure S10A, B). Overexpression of *MPPE1 11‐* significantly promoted gastric cancer cells growth, proliferation and migratory potential *in vitro* (Figure 4D–G and Figure S10C–F), and significantly promoted the tumor growth *in vivo* (Figure 4H–J). Immunohistochemistry assay validated that overexpression of *MPPE1 11‐* resulted in an increase in Ki67 expression (Figure 4K, L), while resulted in a decrease in apoptosis as assayed by TUNEL staining (Figure 4M). Collectively, these data suggested that *MPPE1 11‐* promotes gastric cancer growth *in vitro* and *in vivo*.

**FIGURE 4:**
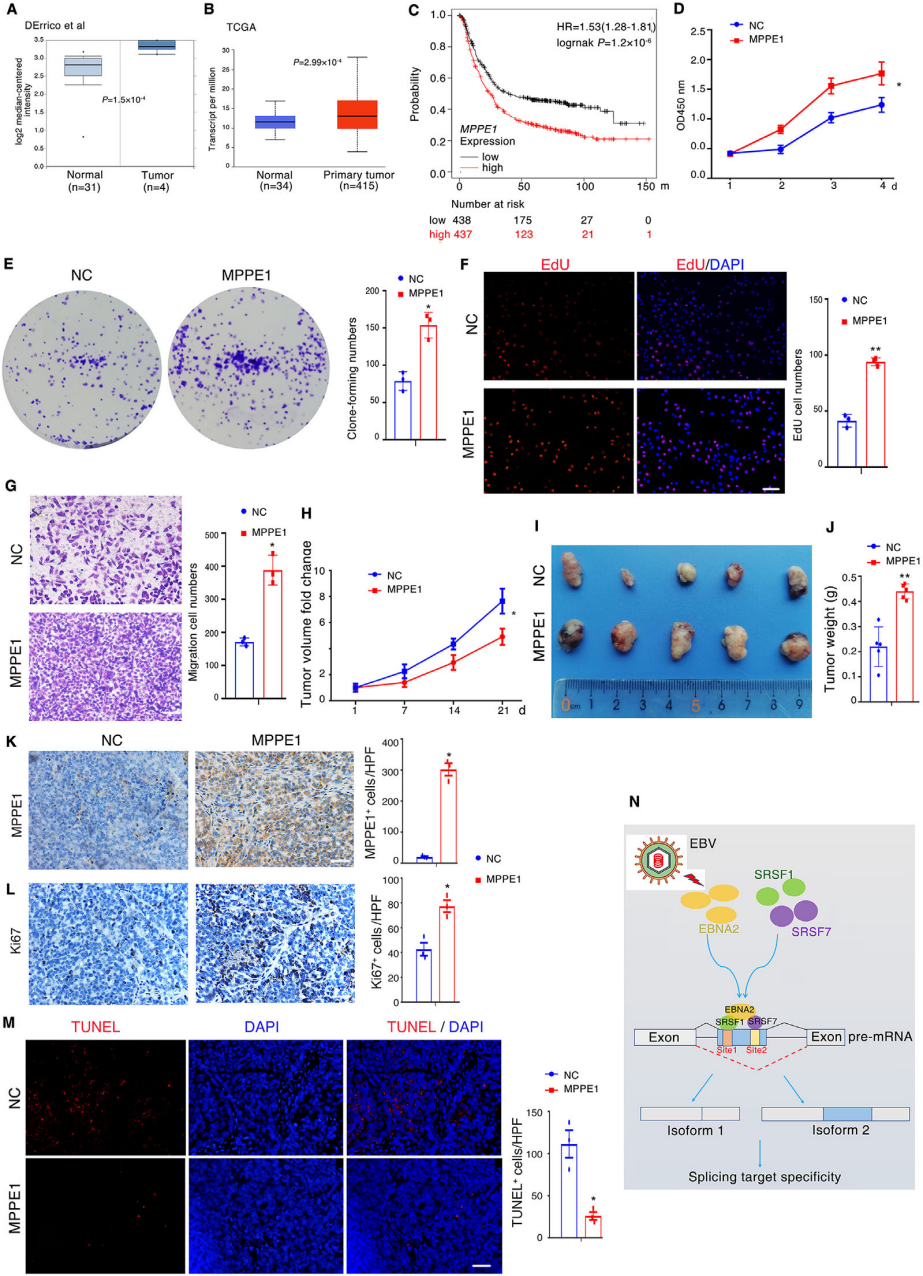
Overexpression of MPPE1 contributes to tumor progression. (A, B) *MPPE1* expression in gastric cancer specimens and non‐cancer controls; the publicly accessible gene expression data of *MPPE1* was obtained from Oncomine database (A) and The Cancer Genome Atlas (TCGA) (B). (C) Kaplan‐Meier overall survival curves according to *MPPE1* expression in patient cohorts in GEO datasets (GSE62254). The percentage of survival patients in high *MPPE1* and low *MPPE1* groups at different time points is presented. (D, F) The proliferation of AGS cells was measured by CCK‐8 (D), Clonogenic (E), and EdU (F) assay after ectopic expression of the *MPPE1* vector. Scale bar, 100 μm. (G) Transwell assay was conducted to test the effect of MPPE1 on the migration ability of AGS cells. A number of cells were counted and shown in the column graph on the right. Data are mean ± SD of three independent experiments. Scale bar, 100 μm. (H) Tumor growth curves of NC and MPPE1‐expressing AGS cells in xenograft tumor growth model. (I) Representative image of xenograft tumors. (J)Tumor weight was smaller in the NC group than that in the MPPE1 group. (K, L) MPPE1 (K) and cell proliferation marker Ki‐67 (L) levels in mouse tumor tissues were assessed with immunohistochemistry. Scale bar, 100 μm. (M) Apoptosis in mouse tumor tissues assessed with TUNEL staining. Significant differences were determined with the Student's *t*‐test. Scale bar, 100 μm. (N) Working model hypothesis for the role of EBNA2 in regulating cellular alternative splicing patterns. In (D–M), NC means the cells were transfected with an empty control vector; MPPE1 means the cells were transfected with “*MPPE1* 11‐ ” vector

In summary, our study shows that EBV EBNA2 interacts with components of the splicing machinery. More than 2000 EBNA2‐affected alternative splicing events are identified by integrating single‐molecule real‐time long‐read sequencing (SMRT) and RNA‐seq. Particularly, EBNA2 regulates *MPPE1* aberrant splicing by recruiting SRSF1 and SRSF7 to its motif in the exon 11, and MPPE1 functions as an oncogene in tumor cells (Figure 4N, proposed working model). Moreover, the ability of EBNA2 in regulating gene‐splicing requires EBNA2 phase separation. These findings suggest that EBNA2‐mediated alternative splicing events play a key role in EBV‐host interactions.

## CONFLICT OF INTEREST

The authors declare that there is no conflict of interest.

## Supporting information

Supporting Information
