# Supplementary material for: Epstein–Barr virus EBNA2 phase separation regulates cancer‐associated alternative RNA splicing patterns
Source: Clin Transl Med. 2021 Aug 9;11(8):e504. doi: 10.1002/ctm2.504 (PMC8351520; doi:10.1002/ctm2.504)
Supplement: Supplementary file 1 — Supporting Information [file CTM2-11-e504-s001.pdf]

Epstein-Barr Virus EBNA2 Phase Separation Regulates Cancer-associated Alternative RNA Splicing Patterns

Supplementary Information

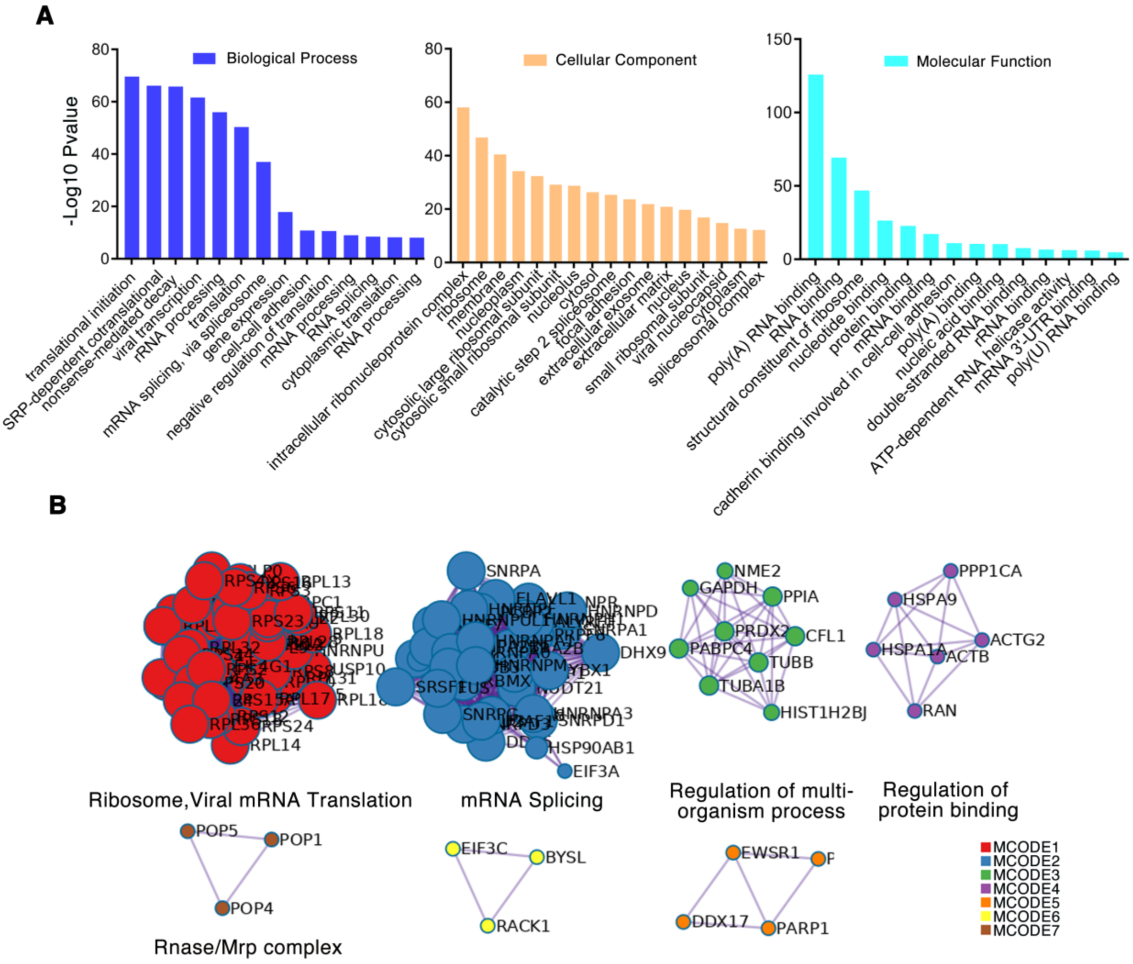

Fig S1. EBNA2 interacts with components of the splicing machinery. (A) Enriched GO categories related to the EBNA2-interacted proteins. The y-axis is enrichment score, and the x-axis is enriched GOs. **(B)** Metascape software (http://metascape.org/) identified seven molecular complex detection (MCODE) among the 193 EBNA2-interacted proteins.

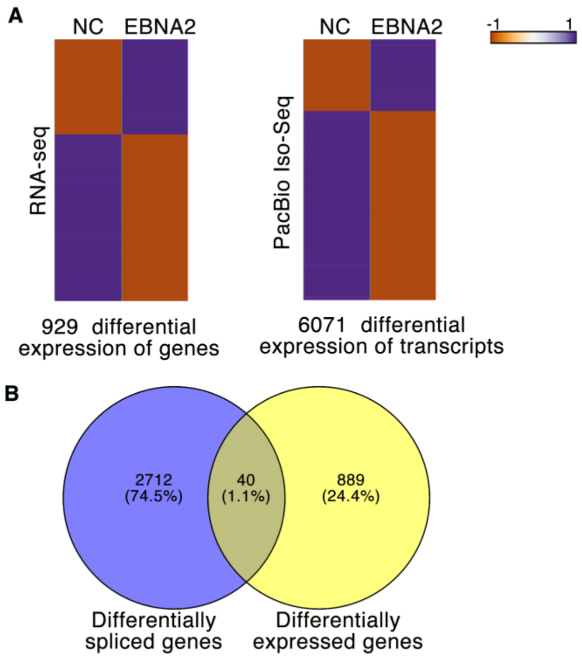

Fig S2. Global landscape of EBNA2-affected alternative splicing events and gene expression. (A) Heatmap of differentially expressed genes and differentially expressed transcripts between NC and EBNA2-overexpression HEK293 cells identified by RNA-seq (left) and PacBio Iso-seq (right). **(B)** Venn comparison plot between differentially spliced genes with differentially expressed genes induced by EBNA2 overexpression in HEK293 cells.

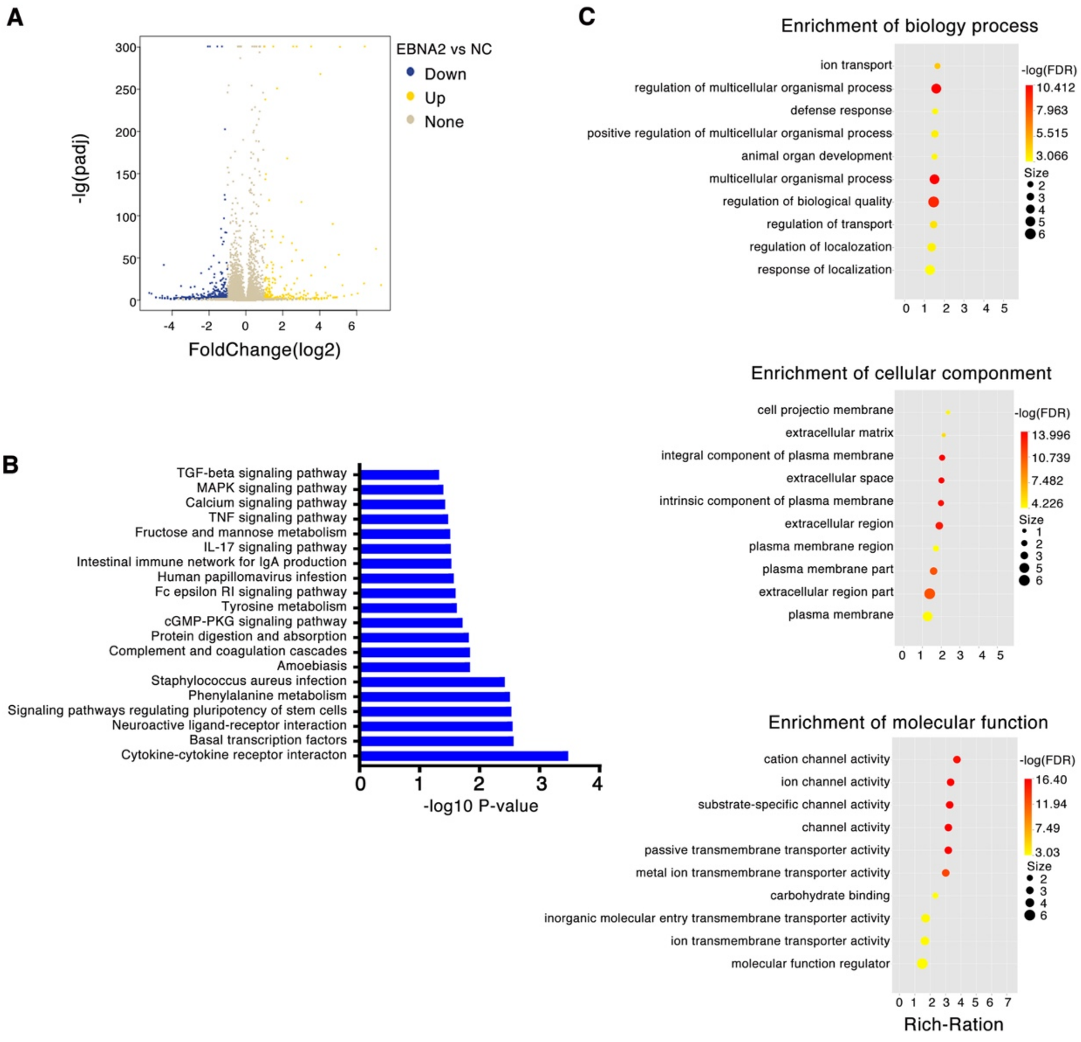

Fig S3. Function analysis of the alternative splicing genes induced by EBNA2 expression. (A) Volcano map presents the EBNA2-regulated differentially spliced genes. **(B)** KEGG pathway was used to analyze the pathways related to the EBNA2-regulated differentially spliced genes. **(C)** GO enrichment analysis was used to analyze the biological functions of the EBNA2-regulated differentially spliced genes. The category includes biological process, cellular component and molecular function.

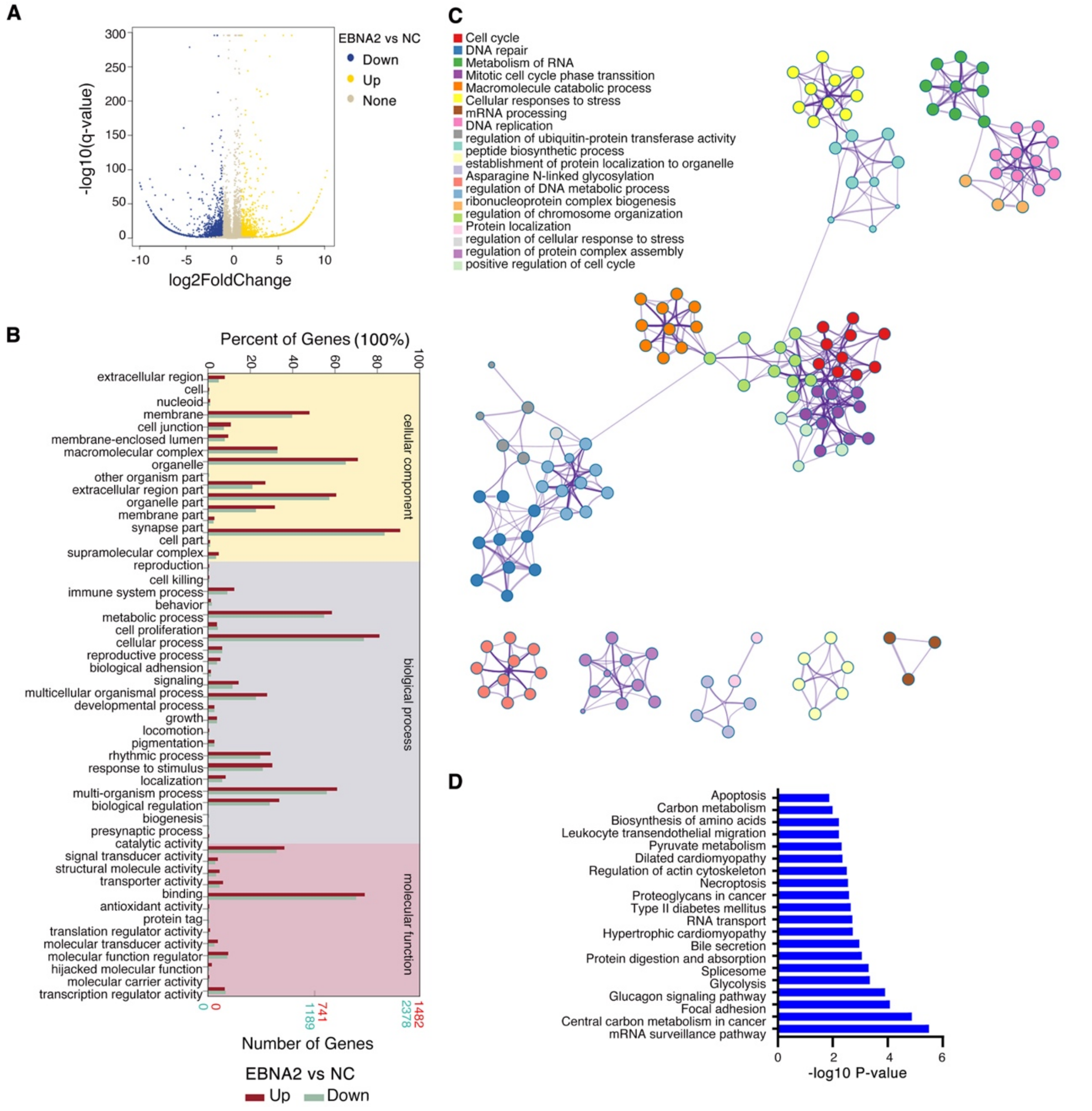

Fig S4. Function analysis of the differentially expressed genes induced by EBNA2 expression. **(A)** Volcano map presents the EBNA2-regulated differentially expressed genes. **(B)** GO enrichment analysis was used to analyze the biological functions of the EBNA2-regulated differentially expressed genes. The category includes biological process, cellular component and molecular function. **(C)** Association of the gene network of EBNA2-regulated differentially expressed genes. **(D)** KEGG pathway was used to analyze the pathways related to the EBNA2-regulated differentially expressed genes.

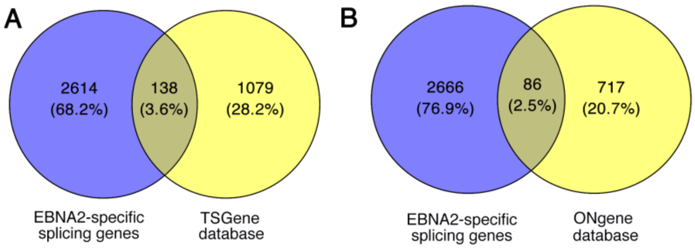

Fig S5. Bioinformatics predicts which genes regulated by EBNA2 are oncogenes or tumor suppressor genes by TSGene **(A)** and ONgene database **(B)**.

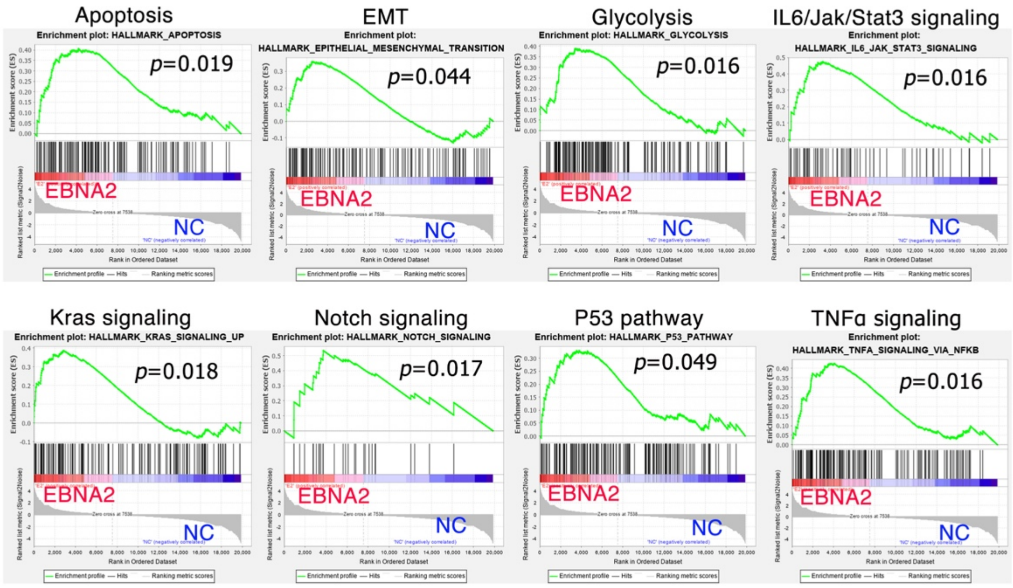

Fig S6. GSEA analysis (http://www.gsea-msigdb.org) showed the multiple tumor-related signal pathways gene sets were enriched in the EBNA2-overexpressed cells comparing to NC cells.

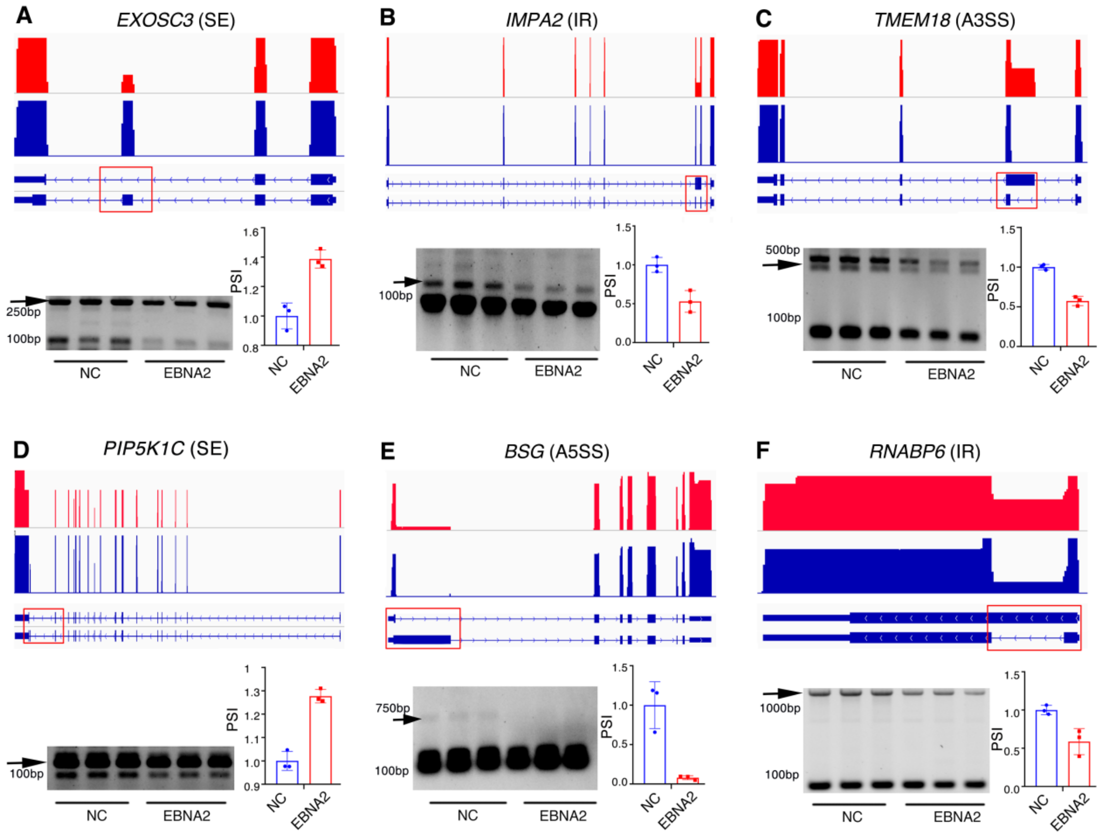

Fig S7. Identification of EBNA2-regulated alternative splicing events for six different genes. *Up panel,* normalized sequencing coverage data are shown in each panel. Maps of EBNA2-specific isoforms shown with exon arrangement and alternative splicing sites highlighted (red rectangle). Scale is same in each track. Relative expression for each gene was calculated based on the normalized RNA-seq read count. *Bottom panel*, a black arrow marks the position of the EBNA2-regulated alternative isoform. RT-PCR was performed on 3 biological replicates, and relative expressions for the isoforms are indicated in the right.

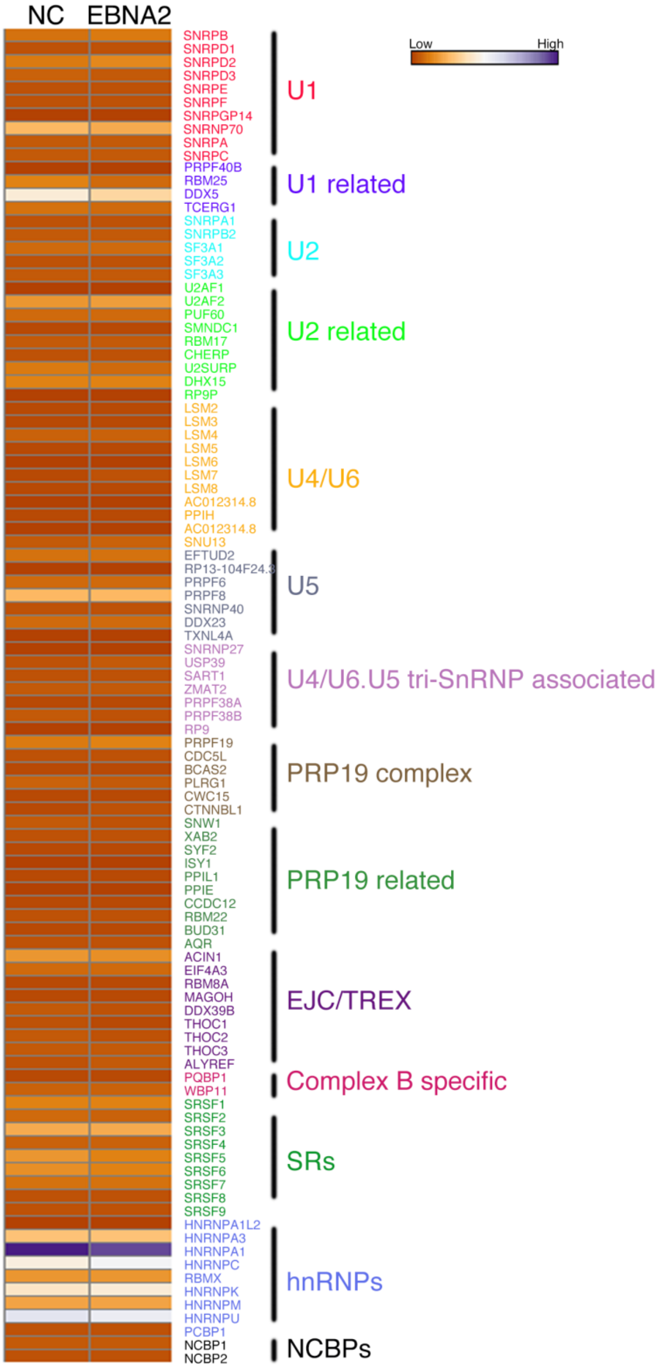

Fig S8. Over-expression of EBNA2 had no effect on the mRNA expression levels of cellular spliceosome components. Heatmap of clustering of expressed genes that involved in the splicing pathway in HEK293 cells (NC vs EBNA2-overexpression). The mRNA levels of those genes are from the RNA-seq data.

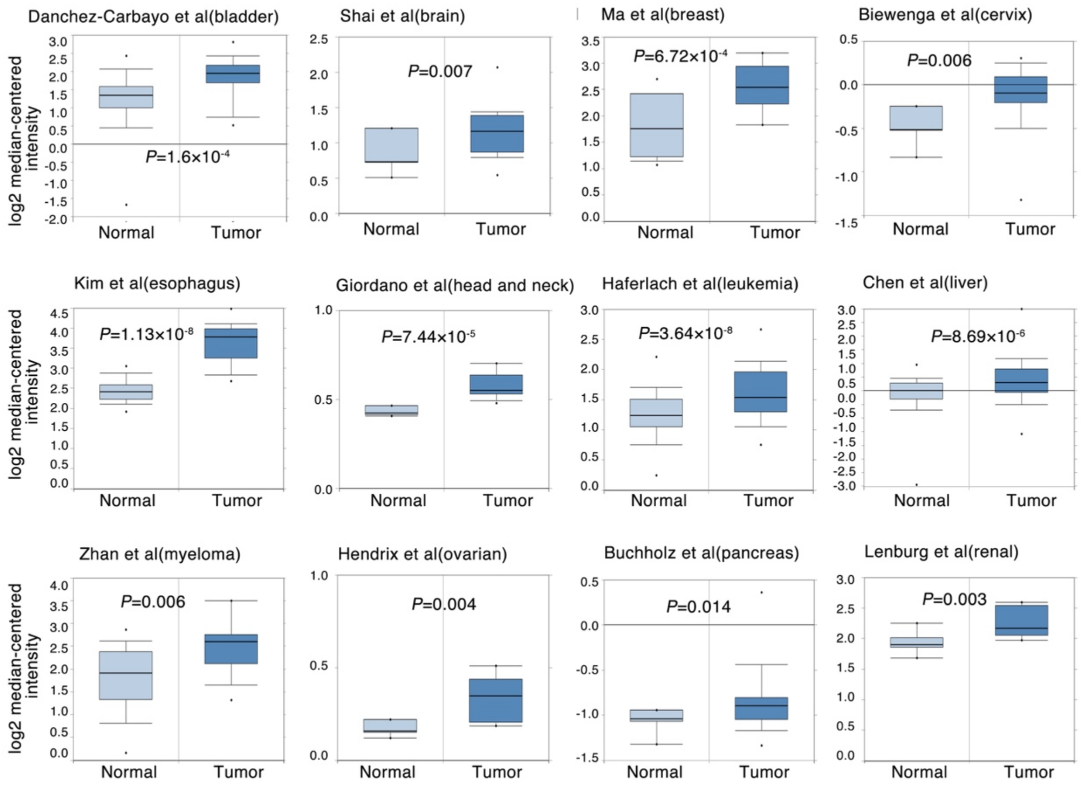

Fig S9. Relative mRNA levels of *MPPE1* analyzed in multiple normal tissues and corresponding carcinoma tissues using Oncomine database (http://www.omcomine.org).

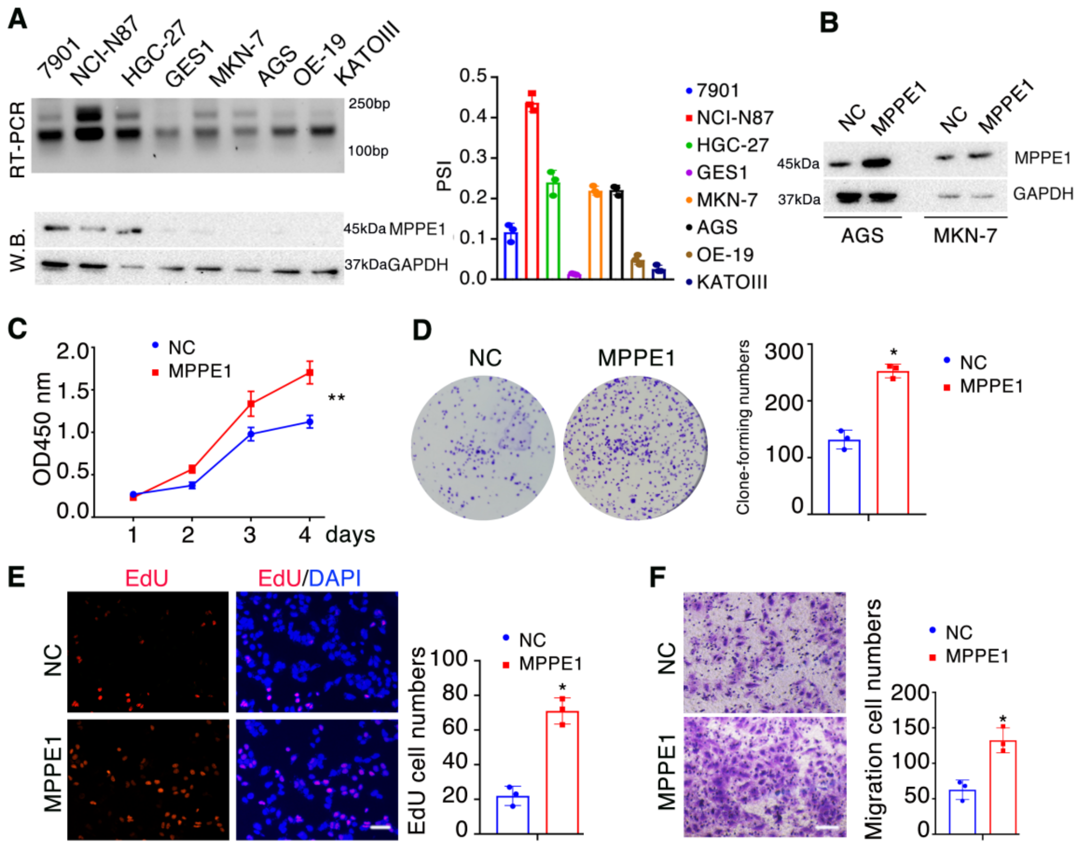

Fig S10. Overexpression of MPPE1 contributes to tumor progression *in vitro*. (A-B) Endogenous MPPE1 expression levels of mRNA (up) and protein (bottom) in multiple gastric cancer cells by RT-PCR and Western blotting. Protein levels are quantified in the right panel. **(B)** Ectopic expression of MPPE1 in AGS and MKN-7 cells and assayed by Western blotting. **(C-E)** The proliferation of MKN-7 cells was measured by CCK-8 **(C)**, Clonogenic **(D)** and EdU **(E)** assay after ectopic expression of MPPE1. Number of cell clones were counted and shown in the column graph on the right panel. Scale bar, 100 µm. **(F)** Transwell assay was conducted to test the effect of MPPE1 on the migration ability of MKN-7 cells. Number of cells were counted and shown in the column graph on the right. Data are mean ± SD of three independent experiments. Scale bar, 100 µm.

Table S1. Interacting proteins of EBNA2 were identified by liquid chromatography mass spectrometry (LC-MS)/MS

Accession	Description	Score	Coverage	# Proteins	# Unique Peptides	# Peptides	# PSMs	# AAs	MW [kDa]	calc. pI
A0A087WTT1	Polyadenylate-binding protein OS=Homo sapiens OX=9606 GN=PABPC1 PE=1 SV=1 - [A0A087WTT1_HUMAN]	23.41	23.18	20	7	11	11	522	58.5	9.26
C9JUE0	Protein TFG (Fragment) OS=Homo sapiens OX=9606 GN=TFG PE=1 SV=1 - [C9JUE0_HUMAN]	21.73	48.41	7	5	5	7	157	17.7	5.05
Q08211	ATP-dependent RNA helicase A OS=Homo sapiens OX=9606 GN=DHX9 PE=1 SV=4 - [DHX9_HUMAN]	20.78	10.55	2	10	10	10	1270	140.9	6.84
Q13283	Ras GTPase-activating protein-binding protein 1 OS=Homo sapiens OX=9606 GN=G3BP1 PE=1 SV=1 - [G3BP1_HUMAN]	20.32	18.03	8	7	7	8	466	52.1	5.52
P22626-2	Isoform A2 of Heterogeneous nuclear ribonucleoproteins A2/B1 OS=Homo sapiens OX=9606 GN=HNRNPA2B1 - [ROA2_HUMAN]	19.49	29.62	3	9	11	13	341	36.0	8.65
P0DMV8-2	Isoform 2 of Heat shock 70 kDa protein 1A OS=Homo sapiens OX=9606 GN=HSPA1A - [HS71A_HUMAN]	17.37	13.82	7	7	8	9	586	63.9	5.71
O60506-4	Isoform 4 of Heterogeneous nuclear ribonucleoprotein Q OS=Homo sapiens OX=9606 GN=SYNCRIP - [HNRPQ_HUMAN]	17.24	18.41	7	6	8	8	527	58.7	7.56
F8VZ49	Heterogeneous nuclear ribonucleoprotein A1 (Fragment) OS=Homo sapiens OX=9606 GN=HNRNPA1 PE=1 SV=1 - [F8VZ49_HUMAN]	16.46	33.77	10	5	7	9	231	25.7	8.27
B1ANR0	Polyadenylate-binding protein OS=Homo sapiens OX=9606 GN=PABPC4 PE=1 SV=1 - [B1ANR0_HUMAN]	16.21	13.50	9	3	7	7	615	67.9	9.45
P62241	40S ribosomal protein S8 OS=Homo sapiens OX=9606 GN=RPS8 PE=1 SV=2 - [RS8_HUMAN]	15.67	40.38	2	7	7	7	208	24.2	10.32
G3V153	Caprin-1 OS=Homo sapiens OX=9606 GN=CAPRIN1 PE=1 SV=1 - [G3V153_HUMAN]	15.48	10.83	4	6	6	7	628	70.3	5.02
A0A0D9SFB3	ATP-dependent RNA helicase DDX3X OS=Homo sapiens OX=9606 GN=DDX3X PE=1 SV=1 - [A0A0D9SFB3_HUMAN]	14.47	10.63	12	4	5	5	640	70.8	7.36
P23396	40S ribosomal protein S3 OS=Homo sapiens OX=9606 GN=RPS3 PE=1 SV=2 - [RS3_HUMAN]	13.92	32.51	14	6	6	6	243	26.7	9.66
P17844-2	Isoform 2 of Probable ATP-dependent RNA helicase DDX5 OS=Homo sapiens OX=9606 GN=DDX5 - [DDX5_HUMAN]	13.13	13.83	6	4	7	7	535	60.5	9.07
A0A1W2PP35	Heterogeneous nuclear ribonucleoprotein U (Fragment) OS=Homo sapiens OX=9606 GN=HNRNPU PE=1 SV=1 - [A0A1W2PP35_HUM	12.80	10.85	16	7	7	7	728	80.6	5.74
Q96PK6	RNA-binding protein 14 OS=Homo sapiens OX=9606 GN=RBM14 PE=1 SV=2 - [RBM14_HUMAN]	12.24	7.62	8	5	5	6	669	69.4	9.67
E9PKE3	Heat shock cognate 71 kDa protein OS=Homo sapiens OX=9606 GN=HSPA8 PE=1 SV=1 - [E9PKE3_HUMAN]	12.24	8.93	15	5	6	7	627	68.8	5.52
Q01844-2	Isoform EWS-B of RNA-binding protein EWS OS=Homo sapiens OX=9606 GN=EWSR1 - [EWS_HUMAN]	12.04	5.83	9	3	3	4	583	61.2	9.07
B4DT28	Heterogeneous nuclear ribonucleoprotein R OS=Homo sapiens OX=9606 GN=HNRNPR PE=1 SV=1 - [B4DT28_HUMAN]	11.78	16.60	6	6	8	8	494	55.7	9.23
Q92841-1	Isoform 2 of Probable ATP-dependent RNA helicase DDX17 OS=Homo sapiens OX=9606 GN=DDX17 - [DDX17_HUMAN]	11.51	10.62	8	4	7	7	650	72.3	8.59
A0A087X2G1	ATP-dependent RNA helicase DDX1 OS=Homo sapiens OX=9606 GN=DDX1 PE=1 SV=1 - [A0A087X2G1_HUMAN]	11.11	14.11	5	7	7	7	659	73.9	7.75
P51991-2	Isoform 2 of Heterogeneous nuclear ribonucleoprotein A3 OS=Homo sapiens OX=9606 GN=HNRNPA3 - [ROA3_HUMAN]	10.54	16.85	2	4	6	7	356	37.0	8.31
P63244	Receptor of activated protein C kinase 1 OS=Homo sapiens OX=9606 GN=RACK1 PE=1 SV=3 - [RACK1_HUMAN]	10.19	27.13	22	6	6	6	317	35.1	7.69
P25398	40S ribosomal protein S12 OS=Homo sapiens OX=9606 GN=RPS12 PE=1 SV=3 - [RS12_HUMAN]	9.68	37.88	1	4	4	4	132	14.5	7.21
P06899	Histone H2B type 1-J OS=Homo sapiens OX=9606 GN=HIST1H2BJ PE=1 SV=3 - [H2B1J_HUMAN]	9.48	27.78	19	3	3	4	126	13.9	10.32
P35637-2	Isoform Short of RNA-binding protein FUS OS=Homo sapiens OX=9606 GN=FUS - [FUS_HUMAN]	9.34	6.67	3	1	3	4	525	53.3	9.36
D6R9P3	Heterogeneous nuclear ribonucleoprotein A/B OS=Homo sapiens OX=9606 GN=HNRNPAB PE=1 SV=1 - [D6R9P3_HUMAN]	8.96	17.86	7	4	5	5	280	30.3	7.91
H0YEN5	40S ribosomal protein S2 (Fragment) OS=Homo sapiens OX=9606 GN=RPS2 PE=1 SV=1 - [H0YEN5_HUMAN]	8.68	27.69	7	4	4	5	195	21.1	9.83
P62701	40S ribosomal protein S4, X isoform OS=Homo sapiens OX=9606 GN=RPS4X PE=1 SV=2 - [RS4X_HUMAN]	8.63	25.48	4	6	6	6	263	29.6	10.15
O14979-3	Isoform 3 of Heterogeneous nuclear ribonucleoprotein D-like OS=Homo sapiens OX=9606 GN=HNRNPDL - [HNRDL_HUMAN]	8.58	25.00	4	4	5	5	244	27.2	8.65
P62269	40S ribosomal protein S18 OS=Homo sapiens OX=9606 GN=RPS18 PE=1 SV=3 - [RS18_HUMAN]	8.55	30.92	4	5	5	5	152	17.7	10.99
Q13151	Heterogeneous nuclear ribonucleoprotein A0 OS=Homo sapiens OX=9606 GN=HNRNPA0 PE=1 SV=1 - [ROA0_HUMAN]	8.49	14.43	1	2	4	5	305	30.8	9.29
Q9NZI8	Insulin-like growth factor 2 mRNA-binding protein 1 OS=Homo sapiens OX=9606 GN=IGF2BP1 PE=1 SV=2 - [IF2B1_HUMAN]	8.23	11.44	8	4	5	5	577	63.4	9.20
H3BR27	RNA-binding motif protein, X chromosome OS=Homo sapiens OX=9606 GN=RBMX PE=1 SV=1 - [H3BR27_HUMAN]	7.94	44.87	11	3	3	3	78	8.6	5.49
J3KRB3	60S ribosomal protein L17 (Fragment) OS=Homo sapiens OX=9606 GN=RPL17 PE=3 SV=3 - [J3KRB3_HUMAN]	7.83	34.88	13	4	4	4	129	14.9	10.39
P39019	40S ribosomal protein S19 OS=Homo sapiens OX=9606 GN=RPS19 PE=1 SV=2 - [RS19_HUMAN]	7.53	22.07	6	3	3	3	145	16.1	10.32
P20073-2	Isoform 2 of Annexin A7 OS=Homo sapiens OX=9606 GN=ANXA7 - [ANXA7_HUMAN]	7.22	10.94	2	4	4	4	466	50.3	6.61
Q15233-2	Isoform 2 of Non-POU domain-containing octamer-binding protein OS=Homo sapiens OX=9606 GN=NONO - [NONO_HUMAN]	7.17	8.12	6	2	3	3	382	43.8	8.63
C9JXB8	60S ribosomal protein L24 OS=Homo sapiens OX=9606 GN=RPL24 PE=1 SV=1 - [C9JXB8_HUMAN]	7.10	24.79	3	3	3	3	121	14.4	11.31
P62277	40S ribosomal protein S13 OS=Homo sapiens OX=9606 GN=RPS13 PE=1 SV=2 - [RS13_HUMAN]	7.08	29.14	3	5	5	5	151	17.2	10.54
Q71UI9-2	Isoform 2 of Histone H2A.V OS=Homo sapiens OX=9606 GN=H2AFV - [H2AV_HUMAN]	6.75	22.81	25	3	3	3	114	12.1	10.46
Q5ST81	Tubulin beta chain OS=Homo sapiens OX=9606 GN=TUBB PE=1 SV=1 - [Q5ST81_HUMAN]	6.70	11.56	17	4	4	4	372	41.7	4.91
G3V203	60S ribosomal protein L18 OS=Homo sapiens OX=9606 GN=RPL18 PE=1 SV=1 - [G3V203_HUMAN]	6.61	20.12	9	3	3	3	164	18.7	11.59
Q9UN86	Ras GTPase-activating protein-binding protein 2 OS=Homo sapiens OX=9606 GN=G3BP2 PE=1 SV=2 - [G3BP2_HUMAN]	6.56	15.56	12	5	5	5	482	54.1	5.55
G3V576	Heterogeneous nuclear ribonucleoproteins C1/C2 OS=Homo sapiens OX=9606 GN=HNRNPC PE=1 SV=1 - [G3V576_HUMAN]	6.51	14.29	25	3	3	3	231	25.2	9.82
P16403	Histone H1.2 OS=Homo sapiens OX=9606 GN=HIST1H1C PE=1 SV=2 - [H12_HUMAN]	6.49	15.49	5	3	3	3	213	21.4	10.93
P08238	Heat shock protein HSP 90-beta OS=Homo sapiens OX=9606 GN=HSP90AB1 PE=1 SV=4 - [HS90B_HUMAN]	6.48	6.35	5	3	3	3	724	83.2	5.03
I3L3P7	40S ribosomal protein S15a OS=Homo sapiens OX=9606 GN=RPS15A PE=1 SV=1 - [I3L3P7_HUMAN]	6.39	40.00	8	4	4	4	100	11.5	10.15
E9PCY7	Heterogeneous nuclear ribonucleoprotein H OS=Homo sapiens OX=9606 GN=HNRNPH1 PE=1 SV=1 - [E9PCY7_HUMAN]	6.38	9.56	21	3	3	3	429	47.1	6.34
P46781	40S ribosomal protein S9 OS=Homo sapiens OX=9606 GN=RPS9 PE=1 SV=3 - [RS9_HUMAN]	6.30	14.95	3	3	3	3	194	22.6	10.65
M0QYT0	Uncharacterized protein (Fragment) OS=Homo sapiens OX=9606 PE=1 SV=1 - [M0QYT0_HUMAN]	6.20	12.77	5	3	3	3	321	36.0	7.42
C9JD32	60S ribosomal protein L23 (Fragment) OS=Homo sapiens OX=9606 GN=RPL23 PE=1 SV=1 - [C9JD32_HUMAN]	6.13	38.46	4	2	2	2	91	9.7	11.49
P68363	Tubulin alpha-1B chain OS=Homo sapiens OX=9606 GN=TUBA1B PE=1 SV=1 - [TBA1B_HUMAN]	6.00	12.64	20	4	4	4	451	50.1	5.06
H0YA96	Heterogeneous nuclear ribonucleoprotein D0 (Fragment) OS=Homo sapiens OX=9606 GN=HNRNPD PE=1 SV=1 - [H0YA96_HUMAN]	5.94	18.10	10	3	4	4	210	23.9	9.58
Q9UKV8-2	Isoform 2 of Protein argonaute-2 OS=Homo sapiens OX=9606 GN=AGO2 - [AGO2_HUMAN]	5.86	8.00	7	5	5	5	825	93.6	9.26
M0R3F1	Heterogeneous nuclear ribonucleoprotein U-like protein 1 (Fragment) OS=Homo sapiens OX=9606 GN=HNRNPUL1 PE=1 SV=1 - [M0R	5.81	3.90	8	2	2	2	641	71.7	8.98
Q07666-2	Isoform 2 of KH domain-containing, RNA-binding, signal transduction-associated protein 1 OS=Homo sapiens OX=9606 GN=KHDRBS1	5.69	7.89	6	4	4	4	418	45.8	7.58
A6NLN1	Polypyrimidine tract binding protein 1, isoform CRA_b OS=Homo sapiens OX=9606 GN=PTBP1 PE=1 SV=4 - [A6NLN1_HUMAN]	5.49	10.25	8	3	3	3	527	56.5	9.38
D6RB09	40S ribosomal protein S3a (Fragment) OS=Homo sapiens OX=9606 GN=RPS3A PE=1 SV=8 - [D6RB09_HUMAN]	5.39	18.65	11	3	3	3	193	22.4	9.76
P61626	Lysozyme C OS=Homo sapiens OX=9606 GN=LYZ PE=1 SV=1 - [LYSC_HUMAN]	5.26	33.11	3	3	3	3	148	16.5	9.16
E9PKG1	Protein arginine N-methyltransferase 1 OS=Homo sapiens OX=9606 GN=PRMT1 PE=1 SV=1 - [E9PKG1_HUMAN]	5.17	21.23	10	5	5	5	325	37.7	6.15
G3V210	60S acidic ribosomal protein P0 OS=Homo sapiens OX=9606 GN=RPLP0 PE=1 SV=1 - [G3V210_HUMAN]	5.08	13.25	12	2	2	2	166	18.3	9.63
Q9Y6M1-1	Isoform 2 of Insulin-like growth factor 2 mRNA-binding protein 2 OS=Homo sapiens OX=9606 GN=IGF2BP2 - [IF2B2_HUMAN]	4.92	8.09	9	2	3	3	556	61.8	8.25
H7C2W9	60S ribosomal protein L31 (Fragment) OS=Homo sapiens OX=9606 GN=RPL31 PE=1 SV=1 - [H7C2W9_HUMAN]	4.85	21.30	8	2	2	2	108	12.8	11.00
Q9Y3I0	tRNA-splicing ligase RtcB homolog OS=Homo sapiens OX=9606 GN=RTCB PE=1 SV=1 - [RTCB_HUMAN]	4.69	5.74	1	2	2	2	505	55.2	7.23
E9PLL6	60S ribosomal protein L27a OS=Homo sapiens OX=9606 GN=RPL27A PE=1 SV=1 - [E9PLL6_HUMAN]	4.60	26.85	4	3	3	3	108	12.2	11.46
Q16629-3	Isoform 3 of Serine/arginine-rich splicing factor 7 OS=Homo sapiens OX=9606 GN=SRSF7 - [SRSF7_HUMAN]	4.57	22.73	10	3	3	3	132	15.2	9.63
P30050	60S ribosomal protein L12 OS=Homo sapiens OX=9606 GN=RPL12 PE=1 SV=1 - [RL12_HUMAN]	4.55	33.94	6	4	4	4	165	17.8	9.42
Q96PE1-2	Isoform 2 of Adhesion G protein-coupled receptor A2 OS=Homo sapiens OX=9606 GN=ADGRA2 - [AGRA2_HUMAN]	4.54	2.68	2	1	1	1	1121	119.8	8.35
A0A1W2PQ43	Bcl-2-associated transcription factor 1 OS=Homo sapiens OX=9606 GN=BCLAF1 PE=1 SV=1 - [A0A1W2PQ43_HUMAN]	4.32	3.84	10	2	2	2	703	80.6	10.10
P62280	40S ribosomal protein S11 OS=Homo sapiens OX=9606 GN=RPS11 PE=1 SV=3 - [RS11_HUMAN]	4.30	34.18	4	5	5	5	158	18.4	10.30
Q07157-2	Isoform Short of Tight junction protein ZO-1 OS=Homo sapiens OX=9606 GN=TJP1 - [ZO1_HUMAN]	4.22	1.92	7	2	2	2	1668	186.9	6.79
P83881	60S ribosomal protein L36a OS=Homo sapiens OX=9606 GN=RPL36A PE=1 SV=2 - [RL36A_HUMAN]	4.05	16.04	6	2	2	2	106	12.4	10.58
E9PFF5	Fragile X mental retardation syndrome-related protein 1 OS=Homo sapiens OX=9606 GN=FXR1 PE=1 SV=1 - [E9PFF5_HUMAN]	3.99	8.37	9	3	3	3	490	55.1	7.49
X6RDA4	Paraspeckle component 1 (Fragment) OS=Homo sapiens OX=9606 GN=PSPC1 PE=1 SV=1 - [X6RDA4_HUMAN]	3.97	11.29	3	2	2	2	248	27.3	5.45
P05141	ADP/ATP translocase 2 OS=Homo sapiens OX=9606 GN=SLC25A5 PE=1 SV=7 - [ADT2_HUMAN]	3.91	11.07	2	3	3	3	298	32.8	9.69
B1AHC9	X-ray repair cross-complementing protein 6 OS=Homo sapiens OX=9606 GN=XRCC6 PE=1 SV=1 - [B1AHC9_HUMAN]	3.74	3.58	3	2	2	2	559	64.2	9.28
Q5VVC9	60S ribosomal protein L11 (Fragment) OS=Homo sapiens OX=9606 GN=RPL11 PE=1 SV=1 - [Q5VVC9_HUMAN]	3.72	10.69	4	1	1	1	131	14.9	8.87
J3KTJ8	60S ribosomal protein L26 (Fragment) OS=Homo sapiens OX=9606 GN=RPL26 PE=4 SV=8 - [J3KTJ8_HUMAN]	3.69	26.04	10	4	4	4	96	11.5	10.90
M0R3D6	60S ribosomal protein L18a (Fragment) OS=Homo sapiens OX=9606 GN=RPL18A PE=1 SV=1 - [M0R3D6_HUMAN]	3.63	25.53	5	3	3	3	141	16.7	10.77
A2A3R5	40S ribosomal protein S6 OS=Homo sapiens OX=9606 GN=RPS6 PE=1 SV=1 - [A2A3R5_HUMAN]	3.58	12.39	3	2	2	2	218	25.0	11.14
H3BQZ7	HCG2044799 OS=Homo sapiens OX=9606 GN=HNRNPUL2-BSCL2 PE=4 SV=1 - [H3BQZ7_HUMAN]	3.53	3.62	2	2	2	2	746	84.6	4.93
H7BY16	Nucleolin (Fragment) OS=Homo sapiens OX=9606 GN=NCL PE=1 SV=8 - [H7BY16_HUMAN]	3.52	6.08	2	2	2	2	296	32.4	5.36
C9J6B6	Eukaryotic translation initiation factor 4 gamma 1 (Fragment) OS=Homo sapiens OX=9606 GN=EIF4G1 PE=1 SV=1 - [C9J6B6_HUMAN	3.35	3.70	15	2	2	2	757	82.4	4.83
A0A0B4J213	60S ribosomal protein L30 OS=Homo sapiens OX=9606 GN=RPL30 PE=1 SV=1 - [A0A0B4J213_HUMAN]	3.18	31.37	4	1	1	1	51	5.6	6.49
F8W7C6	60S ribosomal protein L10 OS=Homo sapiens OX=9606 GN=RPL10 PE=1 SV=2 - [F8W7C6_HUMAN]	3.14	16.56	8	2	2	2	163	18.6	9.95
P62847-2	Isoform 2 of 40S ribosomal protein S24 OS=Homo sapiens OX=9606 GN=RPS24 - [RS24_HUMAN]	3.01	11.54	6	1	1	1	130	15.1	10.89
P62318-2	Isoform 2 of Small nuclear ribonucleoprotein Sm D3 OS=Homo sapiens OX=9606 GN=SNRPD3 - [SMD3_HUMAN]	2.92	8.33	2	1	1	1	120	13.3	8.91
Q01081-4	Isoform 4 of Splicing factor U2AF 35 kDa subunit OS=Homo sapiens OX=9606 GN=U2AF1 - [U2AF1_HUMAN]	2.79	10.78	3	1	1	1	167	19.7	9.06
A0A0C4DGB6	Serum albumin OS=Homo sapiens OX=9606 GN=ALB PE=1 SV=1 - [A0A0C4DGB6_HUMAN]	2.71	7.12	10	3	3	3	604	69.2	6.37
E9PH82	Protein FAM98A OS=Homo sapiens OX=9606 GN=FAM98A PE=1 SV=1 - [E9PH82_HUMAN]	2.71	4.81	3	1	1	1	312	34.4	8.28
E7EPB3	60S ribosomal protein L14 OS=Homo sapiens OX=9606 GN=RPL14 PE=1 SV=1 - [E7EPB3_HUMAN]	2.65	9.68	2	1	1	1	124	14.5	10.21
Q3MHD2	Protein LSM12 homolog OS=Homo sapiens OX=9606 GN=LSM12 PE=1 SV=2 - [LSM12_HUMAN]	2.61	6.67	3	1	1	1	195	21.7	7.74
E5RIP1	40S ribosomal protein S20 OS=Homo sapiens OX=9606 GN=RPS20 PE=1 SV=1 - [E5RIP1_HUMAN]	2.60	23.91	4	1	1	1	46	5.4	9.61
H0YN28	Annexin (Fragment) OS=Homo sapiens OX=9606 GN=ANXA2 PE=1 SV=1 - [H0YN28_HUMAN]	2.59	8.63	7	1	1	1	139	16.1	5.17
P62861	40S ribosomal protein S30 OS=Homo sapiens OX=9606 GN=FAU PE=1 SV=1 - [RS30_HUMAN]	2.53	18.64	2	2	2	2	59	6.6	12.15
E9PLJ3	Cofilin-1 (Fragment) OS=Homo sapiens OX=9606 GN=CFL1 PE=1 SV=1 - [E9PLJ3_HUMAN]	2.53	13.92	8	1	1	1	79	9.1	8.38
C9JFL5	Actin, gamma-enteric smooth muscle (Fragment) OS=Homo sapiens OX=9606 GN=ACTG2 PE=1 SV=8 - [C9JFL5_HUMAN]	2.49	11.11	7	1	1	1	144	16.1	6.27
A0A0G2JQ95	Protein LSM14 homolog A (Fragment) OS=Homo sapiens OX=9606 GN=LSM14A PE=1 SV=1 - [A0A0G2JQ95_HUMAN]	2.48	8.33	6	1	1	1	144	15.9	8.88
Q9BPW8	Protein NipSnap homolog 1 OS=Homo sapiens OX=9606 GN=NIPSNAP1 PE=1 SV=1 - [NIPS1_HUMAN]	2.47	4.93	1	1	1	1	284	33.3	9.31
P06733-2	Isoform MBP-1 of Alpha-enolase OS=Homo sapiens OX=9606 GN=ENO1 - [ENOA_HUMAN]	2.47	5.57	2	1	1	1	341	36.9	6.28
G5E9G0	60S ribosomal protein L3 OS=Homo sapiens OX=9606 GN=RPL3 PE=1 SV=1 - [G5E9G0_HUMAN]	2.46	9.40	6	2	2	2	351	40.2	10.01
M0R055	ELAV-like protein 1 (Fragment) OS=Homo sapiens OX=9606 GN=ELAVL1 PE=1 SV=1 - [M0R055_HUMAN]	2.43	8.27	4	1	1	1	133	14.9	8.16
Q92522	Histone H1x OS=Homo sapiens OX=9606 GN=H1FX PE=1 SV=1 - [H1X_HUMAN]	2.37	5.63	1	1	1	1	213	22.5	10.76
D6RDJ2	40S ribosomal protein S23 OS=Homo sapiens OX=9606 GN=RPS23 PE=1 SV=1 - [D6RDJ2_HUMAN]	2.30	14.55	5	1	1	1	55	6.2	11.12
M0R210	40S ribosomal protein S16 OS=Homo sapiens OX=9606 GN=RPS16 PE=1 SV=1 - [M0R210_HUMAN]	2.27	17.05	4	2	2	2	129	14.4	10.23
E7ERC8	Protein SEC13 homolog (Fragment) OS=Homo sapiens OX=9606 GN=SEC13 PE=1 SV=1 - [E7ERC8_HUMAN]	2.27	12.79	6	1	1	1	86	9.6	6.51
P46779-4	Isoform 4 of 60S ribosomal protein L28 OS=Homo sapiens OX=9606 GN=RPL28 - [RL28_HUMAN]	2.25	15.94	8	1	1	1	69	7.9	10.70
P14625	Endoplasmin OS=Homo sapiens OX=9606 GN=HSP90B1 PE=1 SV=1 - [ENPL_HUMAN]	2.25	1.74	1	1	1	1	803	92.4	4.84
O60716-24	Isoform 3 of Catenin delta-1 OS=Homo sapiens OX=9606 GN=CTNND1 - [CTND1_HUMAN]	2.25	5.43	33	3	3	3	811	90.2	7.15
Q14694	Ubiquitin carboxyl-terminal hydrolase 10 OS=Homo sapiens OX=9606 GN=USP10 PE=1 SV=2 - [UBP10_HUMAN]	2.23	2.51	5	2	2	2	798	87.1	5.31
P62081	40S ribosomal protein S7 OS=Homo sapiens OX=9606 GN=RPS7 PE=1 SV=1 - [RS7_HUMAN]	2.23	4.64	1	1	1	1	194	22.1	10.10
G5E9R0	Actin, cytoplasmic 1 OS=Homo sapiens OX=9606 GN=ACTB PE=1 SV=1 - [G5E9R0_HUMAN]	2.19	14.40	9	1	1	1	125	13.8	5.49
Q15436-2	Isoform 2 of Protein transport protein Sec23A OS=Homo sapiens OX=9606 GN=SEC23A - [SC23A_HUMAN]	2.17	3.20	3	1	1	1	563	63.1	7.74
A0A024R1R8	HCG2014768, isoform CRA_a OS=Homo sapiens OX=9606 GN=hCG_2014768 PE=4 SV=1 - [A0A024R1R8_HUMAN]	2.13	14.06	2	1	1	1	64	7.1	9.99
H3BM89	60S ribosomal protein L4 OS=Homo sapiens OX=9606 GN=RPL4 PE=1 SV=1 - [H3BM89_HUMAN]	2.13	7.21	2	3	3	3	333	37.6	11.03
P78345	Ribonuclease P protein subunit p38 OS=Homo sapiens OX=9606 GN=RPP38 PE=1 SV=2 - [RPP38_HUMAN]	2.09	3.53	1	1	1	1	283	31.8	9.92
P62854	40S ribosomal protein S26 OS=Homo sapiens OX=9606 GN=RPS26 PE=1 SV=3 - [RS26_HUMAN]	2.08	7.83	2	1	1	1	115	13.0	11.00
P52272-2	Isoform 2 of Heterogeneous nuclear ribonucleoprotein M OS=Homo sapiens OX=9606 GN=HNRNPM - [HNRPM_HUMAN]	2.06	7.09	11	3	3	3	691	73.6	8.82
M0R0I5	Far upstream element-binding protein 2 (Fragment) OS=Homo sapiens OX=9606 GN=KHSRP PE=1 SV=1 - [M0R0I5_HUMAN]	2.04	3.94	3	1	1	1	279	29.4	8.57
Q13595-2	Isoform Short of Transformer-2 protein homolog alpha OS=Homo sapiens OX=9606 GN=TRA2A - [TRA2A_HUMAN]	2.04	7.96	4	1	1	1	113	12.9	9.98
H3BND3	Cleavage and polyadenylation-specificity factor subunit 5 (Fragment) OS=Homo sapiens OX=9606 GN=NUDT21 PE=1 SV=8 - [H3BND	2.02	11.41	3	2	2	2	149	17.1	6.38
A0A024R7W5	YTH domain family, member 3, isoform CRA_a OS=Homo sapiens OX=9606 GN=YTHDF3 PE=1 SV=1 - [A0A024R7W5_HUMAN]	2.01	2.62	5	1	1	1	534	58.3	9.23
A6NIW5	Peroxiredoxin 2, isoform CRA_a OS=Homo sapiens OX=9606 GN=PRDX2 PE=1 SV=2 - [A6NIW5_HUMAN]	2.01	7.35	3	1	1	1	136	15.1	6.13
K7EJT5	60S ribosomal protein L22 (Fragment) OS=Homo sapiens OX=9606 GN=RPL22 PE=1 SV=1 - [K7EJT5_HUMAN]	1.96	23.40	7	1	1	1	47	5.1	9.42
P04406-2	Isoform 2 of Glyceraldehyde-3-phosphate dehydrogenase OS=Homo sapiens OX=9606 GN=GAPDH - [G3P_HUMAN]	1.96	9.56	3	2	2	2	293	31.5	7.61
Q15393	Splicing factor 3B subunit 3 OS=Homo sapiens OX=9606 GN=SF3B3 PE=1 SV=4 - [SF3B3_HUMAN]	1.92	1.15	1	1	1	1	1217	135.5	5.26

Q5T2N8	ATPase family AAA domain-containing protein 3C OS=Homo sapiens OX=9606 GN=ATAD3C PE=2 SV=2 - [ATD3C_HUMAN]	1.88	4.14	8	2	2	2	411	46.4	9.31
Q14247-3	Isoform 3 of Src substrate cortactin OS=Homo sapiens OX=9606 GN=CTTN - [SRC8_HUMAN]	1.88	2.73	3	2	2	2	513	57.4	5.33
E7EX53	Ribosomal protein L15 (Fragment) OS=Homo sapiens OX=9606 GN=RPL15 PE=1 SV=1 - [E7EX53_HUMAN]	1.85	15.79	6	2	2	2	133	15.7	11.00
A0A0J9YX35	Immunoglobulin heavy variable 3-64D OS=Homo sapiens OX=9606 GN=IGHV3-64D PE=3 SV=1 - [HV64D_HUMAN]	1.83	9.40	1	1	1	1	117	12.8	7.85
J3QSB4	60S ribosomal protein L13 (Fragment) OS=Homo sapiens OX=9606 GN=RPL13 PE=1 SV=1 - [J3QSB4_HUMAN]	1.83	6.35	3	1	1	1	126	14.7	12.12
J3KSR8	Serine/arginine-rich-splicing factor 1 (Fragment) OS=Homo sapiens OX=9606 GN=SRSF1 PE=1 SV=1 - [J3KSR8_HUMAN]	1.83	6.99	5	1	1	1	143	16.4	11.44
Q13263-2	Isoform 2 of Transcription intermediary factor 1-beta OS=Homo sapiens OX=9606 GN=TRIM28 - [TIF1B_HUMAN]	1.81	2.92	5	2	2	2	753	79.4	5.99
F8WE65	Peptidyl-prolyl cis-trans isomerase OS=Homo sapiens OX=9606 GN=PPIA PE=1 SV=1 - [F8WE65_HUMAN]	1.81	15.00	4	2	2	2	120	13.0	6.77
Q96I24-2	Isoform 2 of Far upstream element-binding protein 3 OS=Homo sapiens OX=9606 GN=FUBP3 - [FUBP3_HUMAN]	1.81	3.07	5	1	1	1	261	28.5	7.88
Q14152-2	Isoform 2 of Eukaryotic translation initiation factor 3 subunit A OS=Homo sapiens OX=9606 GN=EIF3A - [EIF3A_HUMAN]	1.80	0.96	2	1	1	1	1348	162.5	6.77
B4DV51	GTP-binding nuclear protein Ran OS=Homo sapiens OX=9606 GN=RAN PE=1 SV=1 - [B4DV51_HUMAN]	1.79	10.16	5	1	1	1	128	14.7	6.32
F8W0G4	Poly(rC)-binding protein 2 (Fragment) OS=Homo sapiens OX=9606 GN=PCBP2 PE=1 SV=1 - [F8W0G4_HUMAN]	1.78	8.23	13	1	1	1	158	16.6	7.77
A0A087X0Q4	Immunoglobulin kappa variable 2-40 OS=Homo sapiens OX=9606 GN=IGKV2-40 PE=1 SV=1 - [A0A087X0Q4_HUMAN]	1.76	12.50	8	1	1	1	104	11.4	4.49
M0R0G9	U1 small nuclear ribonucleoprotein A OS=Homo sapiens OX=9606 GN=SNRPA PE=1 SV=1 - [M0R0G9_HUMAN]	1.75	20.69	8	2	2	2	87	10.1	9.60
Q49AN9	SNRPG protein OS=Homo sapiens OX=9606 GN=SNRPG PE=1 SV=1 - [Q49AN9_HUMAN]	1.74	18.75	4	1	1	1	64	7.1	7.18
C9J5V9	Nuclease-sensitive element-binding protein 1 (Fragment) OS=Homo sapiens OX=9606 GN=YBX1 PE=1 SV=1 - [C9J5V9_HUMAN]	1.71	3.70	6	1	1	1	216	23.6	10.26
Q9P258	Protein RCC2 OS=Homo sapiens OX=9606 GN=RCC2 PE=1 SV=2 - [RCC2_HUMAN]	1.70	2.11	1	1	1	1	522	56.0	8.78
Q9Y2W1	Thyroid hormone receptor-associated protein 3 OS=Homo sapiens OX=9606 GN=THRAP3 PE=1 SV=2 - [TR150_HUMAN]	1.70	1.15	1	1	1	1	955	108.6	10.15
P62995-3	Isoform 3 of Transformer-2 protein homolog beta OS=Homo sapiens OX=9606 GN=TRA2B - [TRA2B_HUMAN]	1.68	18.62	3	2	2	2	188	21.9	10.15
P38432	Coilin OS=Homo sapiens OX=9606 GN=COIL PE=1 SV=1 - [COIL_HUMAN]	1.65	1.74	1	1	1	1	576	62.6	9.07
Q9NR30-2	Isoform 2 of Nucleolar RNA helicase 2 OS=Homo sapiens OX=9606 GN=DDX21 - [DDX21_HUMAN]	1.65	1.54	2	1	1	1	715	79.6	9.38
Q99575	Ribonucleases P/MRP protein subunit POP1 OS=Homo sapiens OX=9606 GN=POP1 PE=1 SV=2 - [POP1_HUMAN]	1.64	2.25	1	2	2	2	1024	114.6	9.22
H0YB22	40S ribosomal protein S14 (Fragment) OS=Homo sapiens OX=9606 GN=RPS14 PE=1 SV=1 - [H0YB22_HUMAN]	1.62	8.33	3	1	1	1	120	12.9	9.85
A0A075B6Z2	T cell receptor alpha joining 56 (Fragment) OS=Homo sapiens OX=9606 GN=TRAJ56 PE=4 SV=1 - [A0A075B6Z2_HUMAN]	1.62	38.10	1	1	1	1	21	2.2	10.29
P38646	Stress-70 protein, mitochondrial OS=Homo sapiens OX=9606 GN=HSPA9 PE=1 SV=2 - [GRP75_HUMAN]	1.62	4.57	2	2	2	2	679	73.6	6.16
D3YTB1	60S ribosomal protein L32 (Fragment) OS=Homo sapiens OX=9606 GN=RPL32 PE=1 SV=1 - [D3YTB1_HUMAN]	1.62	7.52	3	1	1	1	133	15.6	11.44
K7EQR9	Interleukin enhancer-binding factor 3 (Fragment) OS=Homo sapiens OX=9606 GN=ILF3 PE=1 SV=1 - [K7EQR9_HUMAN]	1.61	10.00	9	1	1	1	180	19.6	8.27
P38919	Eukaryotic initiation factor 4A-III OS=Homo sapiens OX=9606 GN=EIF4A3 PE=1 SV=4 - [IF4A3_HUMAN]	1.61	2.43	1	1	1	1	411	46.8	6.73
B0QY90	Eukaryotic translation initiation factor 3 subunit L OS=Homo sapiens OX=9606 GN=EIF3L PE=1 SV=1 - [B0QY90_HUMAN]	1.61	3.43	4	1	1	1	466	55.1	8.95
H7BY94	Bystin (Fragment) OS=Homo sapiens OX=9606 GN=BYSL PE=1 SV=1 - [H7BY94_HUMAN]	1.60	4.92	2	1	1	1	183	21.0	9.25
O60673-2	Isoform 2 of DNA polymerase zeta catalytic subunit OS=Homo sapiens OX=9606 GN=REV3L - [REV3L_HUMAN]	0.00	0.69	2	1	1	1	3052	343.9	8.47
Q9Y295	Developmentally-regulated GTP-binding protein 1 OS=Homo sapiens OX=9606 GN=DRG1 PE=1 SV=1 - [DRG1_HUMAN]	0.00	3.27	1	1	1	1	367	40.5	8.90
Q99613-2	Isoform 2 of Eukaryotic translation initiation factor 3 subunit C OS=Homo sapiens OX=9606 GN=EIF3C - [EIF3C_HUMAN]	0.00	1.11	3	1	1	1	903	104.0	5.63
P16401	Histone H1.5 OS=Homo sapiens OX=9606 GN=HIST1H1B PE=1 SV=3 - [H15_HUMAN]	0.00	3.98	1	1	1	1	226	22.6	10.92
P07195	L-lactate dehydrogenase B chain OS=Homo sapiens OX=9606 GN=LDHB PE=1 SV=2 - [LDHB_HUMAN]	0.00	2.99	1	1	1	1	334	36.6	6.05
Q8IZF0	Sodium leak channel non-selective protein OS=Homo sapiens OX=9606 GN=NALCN PE=1 SV=1 - [NALCN_HUMAN]	0.00	0.40	1	1	1	1	1738	200.2	8.68
P22392-2	Isoform 3 of Nucleoside diphosphate kinase B OS=Homo sapiens OX=9606 GN=NME2 - [NDKB_HUMAN]	0.00	6.74	8	1	1	1	267	30.1	8.92
P09874	Poly [ADP-ribose] polymerase 1 OS=Homo sapiens OX=9606 GN=PARP1 PE=1 SV=4 - [PARP1_HUMAN]	0.00	1.28	1	1	1	1	1014	113.0	8.88
Q96HS1-2	Isoform 2 of Serine/threonine-protein phosphatase PGAM5, mitochondrial OS=Homo sapiens OX=9606 GN=PGAM5 - [PGAM5_HUMAN	0.00	3.53	2	1	1	1	255	28.0	7.25
Q969H6	Ribonuclease P/MRP protein subunit POP5 OS=Homo sapiens OX=9606 GN=POP5 PE=1 SV=1 - [POP5_HUMAN]	0.00	7.36	1	1	1	1	163	18.8	7.27
Q6P2Q9	Pre-mRNA-processing-splicing factor 8 OS=Homo sapiens OX=9606 GN=PRPF8 PE=1 SV=2 - [PRP8_HUMAN]	0.00	0.43	1	1	1	1	2335	273.4	8.84
Q00577	Transcriptional activator protein Pur-alpha OS=Homo sapiens OX=9606 GN=PURA PE=1 SV=2 - [PURA_HUMAN]	0.00	2.80	1	1	1	1	322	34.9	6.44
P49207	60S ribosomal protein L34 OS=Homo sapiens OX=9606 GN=RPL34 PE=1 SV=3 - [RL34_HUMAN]	0.00	6.84	1	1	1	1	117	13.3	11.47
P05109	Protein S100-A8 OS=Homo sapiens OX=9606 GN=S100A8 PE=1 SV=1 - [S10A8_HUMAN]	0.00	11.83	1	1	1	1	93	10.8	7.03
Q86V81	THO complex subunit 4 OS=Homo sapiens OX=9606 GN=ALYREF PE=1 SV=3 - [THOC4_HUMAN]	0.00	7.00	2	1	1	1	257	26.9	11.15
B4DY09	Interleukin enhancer-binding factor 2 OS=Homo sapiens OX=9606 GN=ILF2 PE=1 SV=1 - [B4DY09_HUMAN]	0.00	13.07	3	3	3	3	352	38.9	4.94
Q5T8U3	60S ribosomal protein L7a (Fragment) OS=Homo sapiens OX=9606 GN=RPL7A PE=1 SV=1 - [Q5T8U3_HUMAN]	0.00	8.38	2	1	1	1	191	21.5	11.02
J3QSB5	60S ribosomal protein L36 OS=Homo sapiens OX=9606 GN=RPL36 PE=1 SV=1 - [J3QSB5_HUMAN]	0.00	10.64	2	1	1	1	94	10.8	11.60
E9PDU6	Calponin (Fragment) OS=Homo sapiens OX=9606 GN=CNN3 PE=1 SV=1 - [E9PDU6_HUMAN]	0.00	5.95	4	1	1	1	185	20.2	9.42
E7ESE0	60S ribosomal protein L9 (Fragment) OS=Homo sapiens OX=9606 GN=RPL9 PE=1 SV=1 - [E7ESE0_HUMAN]	0.00	11.45	3	1	1	1	166	18.9	10.20
H0Y5V1	Serine/threonine-protein kinase MRCK alpha (Fragment) OS=Homo sapiens OX=9606 GN=CDC42BPA PE=1 SV=1 - [H0Y5V1_HUMAN]	0.00	1.91	8	1	1	2	1048	117.8	7.49
H0YLR3	U2 small nuclear ribonucleoprotein A' (Fragment) OS=Homo sapiens OX=9606 GN=SNRPA1 PE=1 SV=1 - [H0YLR3_HUMAN]	0.00	15.73	3	1	1	1	89	9.5	8.47
F8WJN3	Cleavage and polyadenylation-specificity factor subunit 6 OS=Homo sapiens OX=9606 GN=CPSF6 PE=1 SV=1 - [F8WJN3_HUMAN]	0.00	3.35	4	1	1	1	478	52.2	6.43
H0Y653	Nucleolar protein 56 (Fragment) OS=Homo sapiens OX=9606 GN=NOP56 PE=1 SV=1 - [H0Y653_HUMAN]	0.00	5.02	3	1	1	1	219	24.2	9.44
H0YER7	Apoptosis inhibitor 5 (Fragment) OS=Homo sapiens OX=9606 GN=API5 PE=1 SV=1 - [H0YER7_HUMAN]	0.00	3.74	8	1	1	1	294	33.3	8.07
H0YJB9	RNA transcription, translation and transport factor protein (Fragment) OS=Homo sapiens OX=9606 GN=RTRAF PE=1 SV=1 - [H0YJB	0.00	12.40	3	1	1	1	129	14.7	5.60
E9PMD7	Serine/threonine-protein phosphatase (Fragment) OS=Homo sapiens OX=9606 GN=PPP1CA PE=1 SV=1 - [E9PMD7_HUMAN]	0.00	5.53	9	1	1	1	253	28.9	4.87
Q5QPM2	RNA-binding protein Raly (Fragment) OS=Homo sapiens OX=9606 GN=RALY PE=1 SV=1 - [Q5QPM2_HUMAN]	0.00	12.36	5	1	1	1	89	9.7	9.83
J3QLI9	Small nuclear ribonucleoprotein Sm D1 OS=Homo sapiens OX=9606 GN=SNRPD1 PE=1 SV=1 - [J3QLI9_HUMAN]	0.00	17.33	2	2	2	2	75	8.4	11.84
H7BZF9	Lysine-specific demethylase 5D (Fragment) OS=Homo sapiens OX=9606 GN=KDM5D PE=4 SV=1 - [H7BZF9_HUMAN]	0.00	2.73	10	1	1	1	293	32.2	5.31
J3KTN0	Eukaryotic initiation factor 4A-I (Fragment) OS=Homo sapiens OX=9606 GN=EIF4A1 PE=1 SV=1 - [J3KTN0_HUMAN]	0.00	11.11	13	1	1	1	117	13.9	5.15
K7EQV2	Ribonuclease P protein subunit p29 (Fragment) OS=Homo sapiens OX=9606 GN=POP4 PE=1 SV=1 - [K7EQV2_HUMAN]	0.00	6.00	3	1	1	1	150	17.4	10.33
F8W1N5	Nascent polypeptide-associated complex subunit alpha (Fragment) OS=Homo sapiens OX=9606 GN=NACA PE=1 SV=1 - [F8W1N5_H	0.00	19.72	8	1	1	1	71	7.8	9.63
K7EP28	Transcription activator BRG1 (Fragment) OS=Homo sapiens OX=9606 GN=SMARCA4 PE=1 SV=1 - [K7EP28_HUMAN]	0.00	11.03	8	1	1	1	136	15.7	6.81
C9JB90	Ras-related protein Rab-6B (Fragment) OS=Homo sapiens OX=9606 GN=RAB6B PE=4 SV=1 - [C9JB90_HUMAN]	0.00	22.45	61	1	1	1	49	5.9	4.82
A0A1B0GW42	Heterogeneous nuclear ribonucleoprotein F (Fragment) OS=Homo sapiens OX=9606 GN=HNRNPF PE=1 SV=1 - [A0A1B0GW42_HUMA	0.00	8.16	2	1	1	1	98	11.1	5.47

Table S2. Primers information

**Primers for alternative splicing validation**	**F**	**R**
EXOSC3	CGCAGCCACAACCAATTCAT	TCAACTTTCTGCCAATCTGGAGAAG
HRAS	GGAGCAGATCAAACGGGTGAAGGAC	CTGCGTCAGGAGAGCACACACTTGC
MPPE1	CTGGTTCTCAGTGGCCACACGC	CTTGTCTTACGCTTTCCGAGCAAGT
NADK2	GGTCTGAGGCTTCAGGACCCCAACT	CTCTACCAATTCTCTGTTCAATGGA
PIP5K1C	CGGTGCCTCTGCTGCTGTTGAAGT	ACACGTGCTTCCGTCTCTGTGCCAA
RFX2	ACGAACAGCCTACACCTACAACCCC	CTCAGCCCCATAAACACAGAACGGA
CHMP5	GTGTTTGGGTTTCTTCGCGGCT	CTTTGCAGGACCCTCTCTCATCTTC
DUSP22	TATCGGCAGTGGCTGAAGGAAGAAT	GGCATCCAAGCGAGGTACAGAAACC
IMPA2	CTCAAAGGCCTTGGTTCTGACAGAA	GAAGTGTCTATCACGATGCCGCCT
IRF7	TACACGGAGGAACTGCTGCGG	CACCAGGACCAGGCTCTTCTCCTT
PDCD6	GCTACCGGCTCTCTGACCAGTTCCA	GGCTCTTTCCATGTTGTGCTGCTCT
RANBP6	GCTTTGACAGAGCGCAATGGC	GCAATGATTTAGGGCAGTCTTCAAT
SGTA	GCTTCGAACCTAATGAACAATCCCC	GATGAGGCTGGCCAGGTCGTTCT
SIRT6	GGCAGTCGAGGATGTCGGTGAATTA	GGATGCCAGAGGCAGTGCTGAT
SLC25A22	GAGCTGCTGTGAACTTGACCCTCGT	CATCCTGCAGCTGGATCTTCAGCA
THOC1	CTATTATTTCTCTTGCTATTGGGGG	GTGTCACACTGATCCAAAGGAAGAC
BSG	GTTGGAGGTTGTAGGACCGGCGA	CTGTGACCTCTGTGGCGCTGTCATT
TARS	CCTCGCCAGGTAATGGTAGTTCCAG	GATCCAGATCAATGTCTGCCATGAA
TOPORS	CTTCGGAGGGTAGGCGGAGAAGT	GTCTGTTGCAATTTGCTAGTGCCAG
TYMS	AGTACCTGGGGCAGATCCAACACAT	CCACAGCAACTCCTCCAAAACACCC
FSTL3	GTGGTGTTTGCTGGCTCCAGCAG	GTACATGACGCTCAGGTCCGGGT
STK11	GAAGAAACATCCTCCGGCTGAAGCA	CCAGCCTCACTGCTGCTTGCA
TMEM18	GGTTCGGCGGTTGCTGTGAGA	CTAGACACAGAAAGTGCCCGATCTG
TUBB2A	GTAACAAATATGTACCTCGGGCCAT	GCATGAAGAAGTGCAGGCGAG

**Primers for minigene assay**	F	R
EGFP	GAGGGCGAGGGCGATG	CGGACTTGAAGAAGTCGTGC
RFP	GCGTGATGAACTTCGAGGAC	TCTTCTTCTGCATTACGGGGC

**siRNAs of targeting genes**	**Sequence**
si-*SRSF1*	AGGACATTGAGGACGTGTT
si-*SRSF7*	AGGAGAGTTAGAAAGGGCT
si-NC	TTCTCCGAACGTGTCACGTTT

Materials and Methods

Cell culture, plasmid construction, and transfection with short interfering RNA (siRNA)

The human gastric cancer cell lines MKN-7 and AGS, the EBV-positive B lymphoma cell line Raji, the HEK293 cells were cultured in RPMI-1640 medium supplemented with 10% fetal bovine serum. All cell lines were obtained from ATCC. DNA fragments encoding Flag-MPPE1, GFP-EBNA2, Flag-SRSF1 and Flag-SRSF7 were generated by PCR and cloned into a Flag-tagged (p3xFLAG-CMV-10) or GFP-tagged (pEGFP-N1) empty vector and verified by sequencing. siRNAs were purchased from Ribobio (Guangzhou, China). Plasmids and siRNAs were transfected into cells using Lipofectamine 3000 (Invitrogen). siRNA sequences are listed in **Table S2, Supplementary Information.**

Co-Immunoprecipitation (Co-IP) assay and mass spectrometry

Co-IP assays were performed as described [1]. Briefly, HEK293 cells transfected with GFP-EBNA2 and Flag-SRSF1 or Flag-SRSF7 for 48 h were lysed in lysis buffer plus protease inhibitors (Roche) for 30 min at 4 °C. Cell lysates were incubated with 2 μg Flag or GFP antibody at 4 °C overnight. Protein A/G-Sepharose beads (Millipore) were added, and the mix was incubated for 4 h at 4 °C. The immunocomplexes were subsequently washed with lysis buffer three times and subjected to SDS-PAGE, silver stained and subjected to LC–MS/MS for sequencing and data analysis.

Western blotting

Proteins were analyzed by western blotting according to the standard methods. Briefly, Whole cell lysates were isolated using RIPA buffer according to manufacturer's instructions, and the concentration of protein was determined by the BCA Protein Assay Kit. 30 μg of protein was separated by SDS-PAGE on an 8% gradient gel. After transfer to PVDF membranes, the transferred membranes were subsequently incubated overnight at 4°C with primary antibody and then secondary antibody for 1 h. Bands were visualized and quantitated by using the Chemidoc XRS1 system (Bio-Rad).

Alternative splicing events validation by RT-PCR

2 μg of DNA-free RNA was reversed transcribed with an oligo (dT) primer using the Maxima H Minus Reverse Transcriptase (Thermo) according to the manufacturer's protocol. cDNA was amplified with primers spanning the splicing events predicted by IGV tool, separated on a 1% PAGE gel. Band pixel density was calculated using ImageJ on three biological replicates. The relative band intensity was calculated as the ratio of the alternative splicing RT-PCR product versus the constitutive spliced junction product for each replicate. The primer sequences were listed in the **Table S2, Supplementary Information.**

Immunofluorescence confocal microscopy

Immunofluorescence was performed as previously described with some modifications [2]. Raji cells were fixed with 4% paraformaldehyde in PBS for 1 h, permeabilized with 0.5% Triton X-100 and blocked using normal goat serum. Cells were washed three times with PBS; the primary antibodies were added and incubated at room temperature for 2 h. Alexa Fluor 488-conjugated or 568-conjugated secondary antibodies (Beyotime, Jiangsu, China) were added and incubated for 1 h; then, DAPI was used to stain nuclei for 5 min at RT in the dark. The confocal images were acquired using the Leica TCS SP8 confocal laser scanning microscope.

Cell proliferation assay, migration assay and plate-colony formation assay.

Details of the cell proliferation assay were described previously using the Cell Counting Kit-8 (CCK-8; Biotool, China) [3]. EdU incorporation assay was used to detect proliferation ability of cells, and detailed procedures were followed as previously described [4]. For plate-colony formation assay, 1×10^3 cells were seeded into 6-well plates and cultured in RPMI-1640 medium supplemented with 10% FBS. Colonies were fixed with 4% paraformaldehyde and stained with viola crystalline, then scored using a microscope and Image J software. The migration assay was described previously [1]. 5×10^5 cells were seeded onto the upper chamber in 200 μL of serum-free medium; the lower compartment was filled with 500 μL of RPMI-1640 media supplemented with 10% of FBS. After 24 hours incubation, migrated cells on the lower surface of the filter were fixed and stained using viola crystalline.

PacBio Iso-Seq long-read and RNA-Seq short-read sequencing

HEK293 cells was treated with control and GFP-EBNA2 for 48 h and RNA was extracted, sequenced and analyzed by Annoroad Gene Technology Corporation (Beijing, China). For RNA-seq, mRNA was enriched by polyA selection. RNA concentration of library was measured using Qubit® RNA Assay Kit in Qubit® 3.0 to preliminary quantify and then dilute to 1ng/μl. Insert size was assessed using the Agilent Bioanalyzer 2100 system (Agilent Technologies, CA, USA), and qualified insert size was accurate quantification using StepOnePlus™ Real-Time PCR System (Library valid concentration＞10 nM). Then the cell RNA was sequenced by the Illumina NovaSeq 6000 sequencing service.

For PacBio-Seq, Total RNA samples with RIN value≥8 were used for constructing the cDNA libraries in PacBio sequencing. Using the Clontech SMARTer PCR cDNA Synthesis Kit (Takara Biotechnology, Dalian, China), 4μg RNA is synthesized to cDNA and subsequently amplified to generate double-stranded cDNA. The cDNA was then size selected for <4kb, >4 kb fractions using the BluePippin™ Size Selection System (Sage Science, Beverly, MA， USA). Each SMRTbell library was constructed using 1μg size-selected cDNA with the Pacific Biosciences SMRTbell template prep kit. The binding of SMRT bell templates to polymerases was conducted using the Sequel II Binding Kit, and then primer annealing was performed. Sequencing was carried out on the PacBio Sequel II platform by Annoroad Gene Technology Company (Bejing, China). The RNA-seq and PacBio-Seq raw expression files and details have been deposited in NCBI GEO under accession No. GSE165616.

Minigene assay

The *MPPE1* exon 11 minigene was constructed by amplifying the genomic sequence spanning exon 11 and its flanking 100 bp of the human *MPPE1* gene and cloning into the pGint or pRint vector (Addgene), and mutants in which a specific sequence was deleted or inserted were made based on the primary minigene. Vector pGint was designed to contain an EGFP open reading frame (ORF) divided into two exons by a constitutively spliced intron. *MPPE1* exon 11 and its flanking intron region was amplified with *MPPE1* minigene-F and *MPPE1* minigene-R from genome DNA and inserted into the intron. When exon 11 retained, the EGFP or RFP ORF is disrupted and thus low or no green or red fluorescence is detected. Exon 11 skipping results in a functional EGFP or RFP ORF and expression. The primer sequences were listed in the **Table S2, Supplementary Information.**

Public datasets analysis

The data of *MPPE1* regarding expression levels and survival time were obtained from Oncomine (http://www.omcomine.org), the public TCGA samples were analyzed by the UALCAN database (http://ualcan.path.uab.edu/index.html) and Kaplan–Meier plotter (http://www.kmplot.com/), databases. Search strategies were as previous report [5].

Fluorescence recovery after photo bleaching (FRAP) assay

FRAP were performed as described[6]. Briefly, using a Leica TCS SP8 confocal microscope with 2.4-milliwat laser intensity for bleaching at 37 °C, 63×/1.4 oil immersion objective, and photomultiplier tube detector. EGFP labeled proteins were photo-bleached by 488 nm laser beams at 100% power respectively. Each FRAP experiment involved 2 pre-bleach frames, followed by 1 bleach frames, and recovery was monitored over 50 s. Three droplets were bleached at a time for each experiment. Each data point represented the averaged signal of three regions of interest (ROI) with similar sizes.

TUNEL assay

Terminal deoxynucleotidyl transferase-mediated dUTP nick end labeling (TUNEL) assay was performed using the One-Step TUNEL Apoptosis Assay Kit (Beyotime, Jiangsu, China) according to the manufacturer's recommended protocol.

Immunohistochemistry

Paraffin-embedded sections were cut 4-µm thick, then deparaffinized and rehydrated. MPPE1and Ki67 were detected by immunohistochemical staining as described[7].

Animal experiment

All animal care and euthanasia protocols were approved by the Institutional Animal Care and Use Committee of Central South University (Changsha, China). For the cancer cell xenograft study, 4-week-old nude male mice were implanted into the right flank of mice subcutaneously with 5×10^6 MPPE1-overexpressed (or negative control, NC) AGS cells in 100 µl RPMI-1640 that was mixed with matrigel (1:1). After 4 weeks, the mice were dissected, and the tumor tissue was removed for fixation, dehydration, and photographs.

Statistical Analysis

Statistical analysis was performed using GraphPad Prism 8 and SPSS17. Data were typically expressed as the mean ± SD, and the differences between groups were assessed with the unpaired Student t-test. P-values < 0.05 were considered to reflect statistical significance.

References for Supplementary Information

1. Peng Q, Chen L, Wu W, Wang J, Zheng X, Chen Z, Jiang Q, Han J, Wei L, Wang L, et al. EPH receptor A2 governs a feedback loop that activates Wnt/beta-catenin signaling in gastric cancer. Cell Death Dis 2018, 9:1146.
2. Fan C, Qu H, Xiong F, Tang Y, Tang T, Zhang L, Mo Y, Li X, Guo C, Zhang S, et al. CircARHGAP12 promotes nasopharyngeal carcinoma migration and invasion via ezrin-mediated cytoskeletal remodeling. Cancer Lett 2020, 496:41-56.
3. Ou C, Sun Z, He X, Li X, Fan S, Zheng X, Peng Q, Li G, Li X, Ma J. Targeting YAP1/LINC00152/FSCN1 Signaling Axis Prevents the Progression of Colorectal Cancer. Adv Sci (Weinh) 2020, 7:1901380.
4. Yu Z, Sun Y, She X, Wang Z, Chen S, Deng Z, Zhang Y, Liu Q, Liu Q, Zhao C, et al. SIX3, a tumor suppressor, inhibits astrocytoma tumorigenesis by transcriptional repression of AURKA/B. J Hematol Oncol 2017, 10:115.

5. Wang L, Peng Q, Sai B, Zheng L, Xu J, Yin N, Feng X, Xiang J. Ligand-independent EphB1 signaling mediates TGF-beta-activated CDH2 and promotes lung cancer cell invasion and migration. J Cancer 2020, 11:4123-4131.
6. Peng Q, Wang L, Qin Z, Wang J, Zheng X, Wei L, Zhang X, Zhang X, Liu C, Li Z, et al. Phase Separation of Epstein-Barr Virus EBNA2 and Its Coactivator EBNALP Controls Gene Expression. J Virol 2020, 94.
7. Li Z, Wang J, Zhang X, Liu P, Zhang X, Wang J, Zheng X, Wei L, Peng Q, Liu C, et al. Proinflammatory S100A8 Induces PD-L1 Expression in Macrophages, Mediating Tumor Immune Escape. J Immunol 2020, 204:2589-2599.
